# Scandium—Group 13 Heterobimetallic Methylidene Clusters

**DOI:** 10.1021/acs.inorgchem.5c01520

**Published:** 2025-07-02

**Authors:** Gernot T. L. Zug, Cäcilia Maichle-Mössmer, Reiner Anwander

**Affiliations:** † 9188 Institut für Anorganische Chemie, Eberhard Karls Universität Tübingen, Auf der Morgenstelle 18, 72076 Tübingen, Germany

## Abstract

A straightforward synthesis of Cp*_2_Sc­(AlMe_4_) (Cp* = C_5_Me_5_) applying Cp*_2_ScCl­(thf) and LiAlMe_4_/AlMe_3_ is described. Donor-assisted trimethyltriel exchange gives access to Cp*_2_Sc­(EMe_4_) (E = Ga, In), which represent the first scandium tetramethylgallate and tetramethylindate complexes. Thermal treatment of Cp*_2_Sc­(EMe_4_) (E = Ga, In) in benzene leads to the isolation of methylidene clusters Cp*_6_Sc_4_E_8_(CH_2_)_12_Me_6_ (E = Ga, In). This methyl group deprotonation differs from the benzene activation previously observed for Cp*_2_Y­(EMe_4_) (E = Al, Ga). Treatment of Cp*_2_Sc­(EMe_4_) (E = Al, Ga) with excess GaMe_3_ in benzene at elevated temperatures generated Ga_8_(CH_2_)_12_, following the known reactivities of Cp*_2_Ln­(GaMe_4_) (Ln = Y, Lu) with GaMe_3_. Analogous reactions of scandocene complexes Cp*_2_Sc­(EMe_4_) (E = Al, In) with excess InMe_3_ at elevated temperatures did not yield the putative homoleptic indium methylidene. Instead, the methylidene cluster Cp*_4_Sc_4_In_8_(CH_2_)_12_Me_8_ was isolated, which features half-sandwich scandium fragments exclusively. A reaction of InMe_3_ and HCp* at high temperatures yielded Me_2_InCp*InMe_3_, which might incorporate the missing Cp* ligands in the cluster formations. Rare-earth-metal compounds were analyzed by SC-XRD, ICP-OES and elemental analysis. Compounds Cp*_2_Sc­(EMe_4_) (E = Ga, In) and [Cp*_2_Sc­(ClAlMe_3_)]_2_ were additionally analyzed by ^1^H, ^13^C­{^1^H}, ^45^Sc NMR as well as ^1^H and ^45^Sc variable temperature NMR studies.

## Graphical abstract

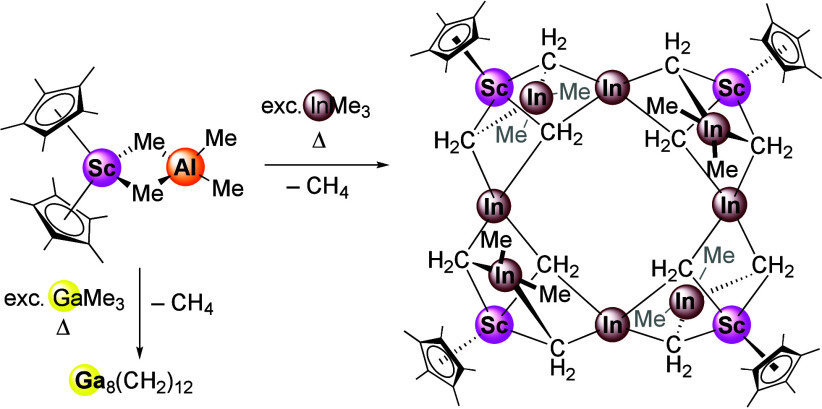

## Introduction

The feasibility of discrete early transition metal methylidene complexes draws on sterically demanding ancillary ligands (kinetic stabilization) and Lewis-acid (specifically group 13 alkyl) stabilization. Tebbe’s reagent, Cp_2_Ti­(CH_2_)­(Cl)­AlMe_2_ (Cp = C_5_H_5_), is a prime example of combining both synthesis approaches.^1^ Unsurprisingly, the synthesis of terminal, unsupported (Lewis acid free) methylidene complexes has posed an overriding challenge. Walking the line V–Ti–Sc, in contrast to the pentavalent mixed imido/methylidene (PN)­V­(=CH_2_)­(NC_6_H_3_ *i*Pr_2_-2,6) and tetravalent methylidene (PN)_2_Ti­(=CH_2_) (PN = (N-(2-(diisopropylphosphino)-4-methylphenyl)-2,4,6-trimethylanilide)), a trivalent terminal scandium methylidene complex has remained elusive.^2^ Naturally, a potentially terminal sandwich scandocene­(III) methylidene fragment would be anionic. On the other hand, Tebbe-like Lewis acid stabilized neutral methylidene complexes with one monoanionic ancillary ligand, like (PNP)­Sc­(CH_2_)­(AlMe_3_)_2_ (PNP = N­[2-P­(*i*Pr)_2_-4-methylphenyl]_2_ (ref 3) or (Cp^R^)­Sc­(CH_2_)­(AlMe_3_)_2_ (ref 4) have been structurally authenticated, while respective half-sandwich complexes devoid of Lewis-acid stabilization form clusters like tetrametallic [(C_5_Me_4_R)­Sc­(μ–CH_2_)]_4_ via thermolysis of [(C_5_Me_4_R)­Sc­(μ–CH_3_)­(CH_3_)]_2_ (R = Me, SiMe_3_).^5^ Thermolysis of neopentyl complex (PNP-Cy)­Sc­(CH_2_ *t*Bu)_2_ (PNP-Cy = 2,5-bis­(dicyclohexylphosphinomethyl)­pyrrolide) generated the methylidene-bridged dimeric scandium complex [(PNP-Cy)­Sc­(μ–CH_2_)]_2_ via a terminal methylidene intermediate, as derived from deuterium labeling experiments and DFT calculations.^6^

Rare-earth-metal methylidenes can be obtained from methyl precursors via methyl ligand deprotonation. The activation of external C–H bonds has been extensively investigated in organo-rare-earth-metal chemistry ever since methane activation by the sandwich compound Cp*_2_LuMe was reported by Watson in 1983 and the application of Cp*_2_ScMe in σ-bond metathesis reactions was highlighted by the group of Bercaw in 1987.^7^ When external C–H bond activation is disfavored in aliphatic solvents, Ln–CH_3_ moieties can engage in internal C–H-bond activation involving, e.g., cyclopentadienyl methyl groups to form tuck-in or tuck-over complexes such as [Cp*Sc­(C_5_Me_4_CH_2_)]_2_.^7b,8^

We have recently shown that there are considerable similarities between rare-earth-metal methyls [Cp*_2_LnMe]_*x*_ and tetramethylaluminates Cp*_2_Ln­(AlMe_4_). Depending on the solvent employed, Cp*_2_Ln­(AlMe_4_) can promote distinct/competitive C–H-bond activation. The yttrocenes Cp*_2_Y­(EMe_4_) (E = Al, Ga) activate (external) benzene to yield phenyl complexes Cp*_2_Y­(EPh_4_) (E = Al, Ga).^9^ On the other hand, Cp*_2_Ln­(GaMe_4_) (Ln = Y, Lu) react at 130 °C in toluene with excess GaMe_3_ to form homoleptic gallium methylidene Ga_8_(CH_2_)_12_.^10^ The latter reaction might involve activation of an internal C–H bond to an active species which catalyzes the reaction of GaMe_3_ to Ga_8_(CH_2_)_12_. In contrast, heating a toluene solution of Cp*_2_Lu­(AlMe_4_) with excess AlMe_3_ does not yield homoleptic aluminum methylidene but methyl aluminum methylidene [MeAl­(CH_2_)]_12_, which could be also accessed by the reaction of Ga_8_(CH_2_)_12_ with AlMe_3_ or a Cp_2_TiCl_2_/AlMe_3_ mixture.^11^ In addition, treatment of Cp*_2_Ln­(GaMe_4_) (Ln = Y, Lu) with two equivalents GaMe_3_ at 130 °C in methylcyclohexane formed methylidene clusters featuring three sandwich rare-earth-metal fragments bound to the cluster core.^10^ Moreover, the reaction of Cp*_2_Lu­(AlMe_4_) with four equivalents AlMe_3_ at 130 °C in toluene-*d*_8_ formed a methylidene cluster, with two sandwich rare-earth-metal fragments bound to the cluster core.^11^ In both cases, the sandwich rare-earth-metal fragment remained intact.

The previous examples of Cp*_2_Ln­(EMe_4_) (E = Al, Ga) did not involve scandium. Overall, the properties of scandium tetramethylaluminate complexes have been explored just scarcely. This is mainly due to the homoleptic precursor Sc­(AlMe_4_)_3_ being stable only below −10 °C, while the congeners of all other rare-earth metals (Y, La–Lu, except Pm, Eu) are stable at ambient temperature.^12^ This stability difference is mainly due to the comparatively small size of scandium (effective radius of hexacoordinate of Sc­(III): 0.745 Å; Lu­(III): 0.861 Å),^13^ entailing distinct properties such as considerably increased Lewis acidity and polarizing power.

We have recently launched a study on how the type of group 13 metal might affect the formation of Lewis-acid stabilized rare-earth-metal complexes with multiply bonded main group fragments/ligands. For example, homoleptic gallates Ln­(GaMe_4_)_3_ (Ln = Ce, Nd, Sm) gave access to terminal imides while the respective aluminates afforded Lewis acid stabilized imides exclusively.^14^ In this work, we report on the synthesis of the first scandium tetramethylgallate and tetramethylindate complexes Cp*_2_Sc­(EMe_4_) (E = Ga, In) using a donor-assisted tetramethylaluminate/tetramethyltrielate exchange protocol.^15^ Such transformations driven by Pearsoǹs HSAB concept routinely suffer from incomplete exchange, which means that the products Cp*_2_Sc­(EMe_4_) (E = Ga, In) still contained residual aluminum. Pursuing the above-mentioned C–H-bond activation chemistry, established previously for larger rare-earth metals, we found that thermal treatment of Cp*_2_Sc­(EMe_4_) (E = Al, Ga, In) led favorably to internal methyl ligand deprotonation rather than external benzene activation. From these reactions, methylidene clusters of the type Cp*_6_Sc_4_E_8_(CH_2_)_12_Me_6_ (E = Ga, In) could be isolated.

## Results and Discussion

### Syntheses of Cp*_2_Sc­(AlMe_4_), [Cp*_2_Sc­(ClAlMe_3_)]_2_, and Cp*_2_ScMe­(thf) (thf = Tetrahydrofuran)

Compound Cp*_2_Sc­(AlMe_4_) (**1-Al**) was previously obtained via the reaction of the temperature-sensitive homoleptic Sc­(AlMe_4_)_3_ with two equivalents KCp*.^12^ As the Sc­(AlMe_4_)_3_-based synthesis is rather elaborate and **1-Al** is stable at ambient temperature, we envisaged an alternative reaction path. Application of a salt-metathesis protocol seemed most feasible, given that the first scandium tetramethylaluminate complex reported, scandocene Cp_2_Sc­(AlMe_4_), was obtained from Cp_2_ScCl and LiAlMe_4_ in toluene.^16^ Accordingly, the known sandwich chloride precursor was obtained from ScCl_3_(thf)_3_ and a slight excess of LiCp* in refluxing toluene (Scheme 1).^7b^ In order to trap evolving THF in the reaction mixture, two equivalents AlMe_3_ were added. The reaction mixture was filtered and volatiles were removed *in vacuo* to yield a reddish powder, depicted as “Cp*_2_ScCl­(thf)”. Without further purification this reddish powder was treated with one equivalent LiAlMe_4_ and three equivalents AlMe_3_ to bind any remaining THF. Filtration and recrystallization from a toluene/*n*-hexane mixture afforded pure Cp*_2_Sc­(AlMe_4_) (**1-Al**) in 72% yield relating to ScCl_3_(thf)_3_. ^1^H and ^45^Sc NMR spectra (δ_Sc_ 92.6 ppm) were in accordance with previously reported data.^12^ Therefore, this synthesis approach represents a more convenient and higher-yielding route to **1-Al** than the Sc­(AlMe_4_)_3_-based one. It should be noted that the group of Evans recently reported on new syntheses toward Cp*_2_ScCl­(thf) and Cp*_2_ScCl using KCp* instead of LiCp* and without the need to remove evolving THF under reduced pressure or the addition of a Lewis acid.^17^

**Scheme 1:**
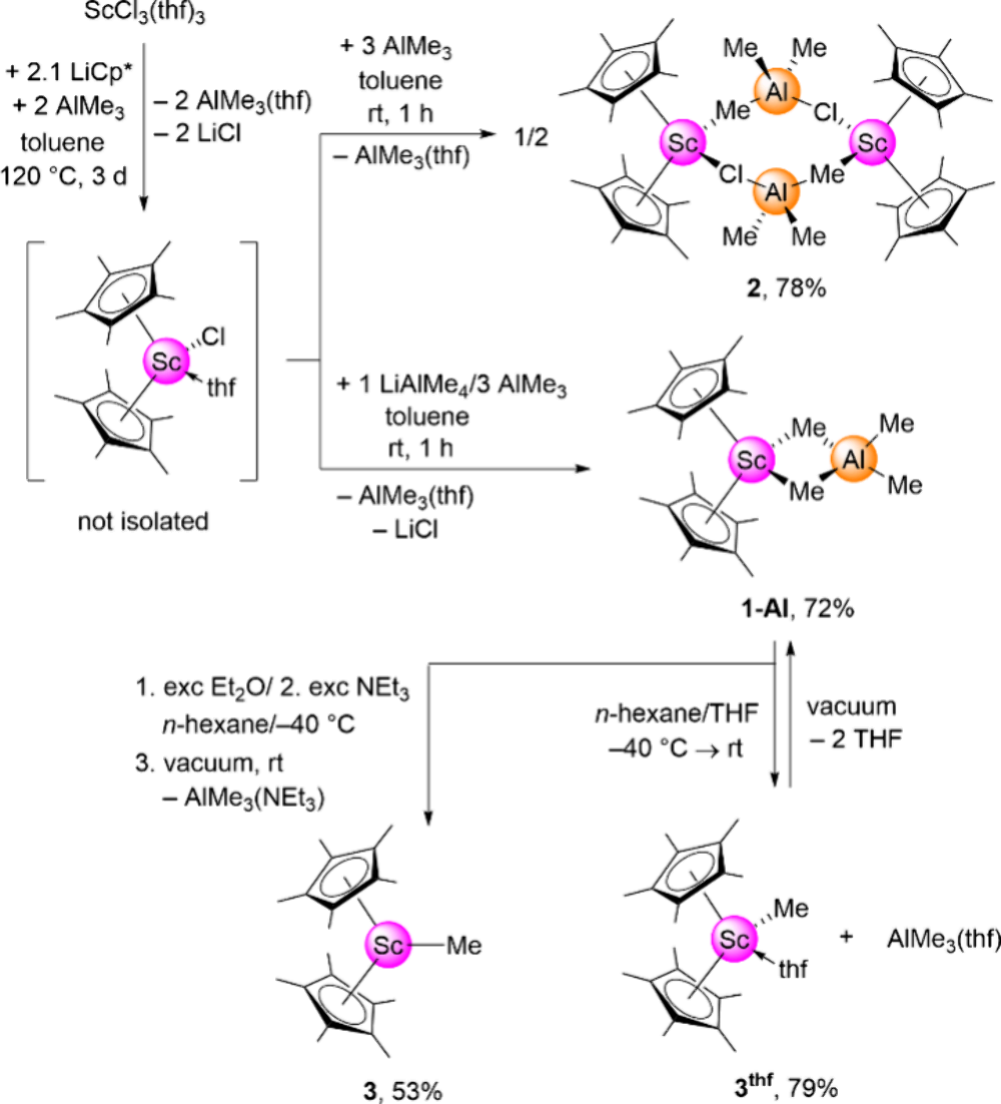
Synthesis of Sandwich Compounds Cp*_2_Sc­(AlMe_4_) (**1-Al**), [Cp*_2_Sc­(ClAlMe_3_)]_2_ (**2**), Cp*_2_ScMe (3), and Cp*_2_ScMe­(thf) (**3** ^**thf**^)

Addition of only AlMe_3_ (without LiAlMe_4_) to crude, reddish “Cp*_2_ScCl­(thf)” gave the mixed chloride/trimethylaluminato complex [Cp*_2_Sc­(ClAlMe_3_)]_2_ (**2**). Dimeric **2** is a rare example featuring such a ligand combination. The only other structurally characterized examples comprise [Cp*_2_Sm­(ClAlMe_3_)]_2_, [Cp*_2_Sm­(ClAlEt_3_)]_2_, Cp*_2_Sm­(ClAl*i*Bu_3_), Cp*_2_Y­(ClAlEt_3_), and Cp*_2_Y­(ClAl*i*Bu_3_) from the group of Evans, as well as Cp^ttt^_2_Nd­(ClAlMe_3_) (Cp^ttt^ = 1,2,4-tri­(*tert*-butyl)­cyclopentadienyl) from the group of Sitzmann.^18^

The ^1^H, ^13^C­{^1^H}, and ^45^Sc NMR spectra of aromatic solutions of **2** show signals of Cp*_2_Sc­(AlMe_4_) (**1-Al**). The prevailing ligand scrambling would produce Cp*_2_ScCl besides Cp*_2_Sc­(AlMe_4_), but any distinct ^1^H NMR signal of the chloride derivative was not observed.^17^ However, the supposed equilibrium of [Cp*_2_Sc­(ClAlMe_3_)]_2_ and Cp*_2_Sc­(AlMe_4_) in solution is further backed by variable temperature ^1^H and ^45^Sc NMR studies of **2** (Figure S5 and S6). The variable temperature ^1^H NMR study of **2** revealed the literature-known signals of Cp*_2_Sc­(AlMe_4_) at low temperatures,^12^ and one additional signal set of [Cp*_2_Sc­(ClAlMe_3_)]_2_. Additionally, as common for sandwich rare-earth-metal aluminates, we suppose the occurrence of a monomer–dimer equilibrium.

Work by Lappert et al. had also revealed that treatment of Cp_2_Sc­(AlMe_4_) with donor molecules like pyridine resulted in tetramethylaluminato cleavage affording donor adducts Cp_2_ScMe­(py) and AlMe_3_(py).^19^ Soon after in 1985, Watson and Parshall reported on the isolation of donor-free Cp*_2_LnMe (Ln = Yb, Lu) by treatment of Cp*_2_Ln­(AlMe_4_) (Ln = Yb, Lu) with diethyl ether, then triethylamine, followed by the removal of volatiles.^20^ According to the latter approach, **1-Al** could be converted into Bercaẁs seminal donor-free scandocene Cp*_2_ScMe (**3**) and obtained in single-crystalline form. Compound **3** was previously obtained from Cp*_2_ScCl and LiMe in toluene/Et_2_O. The new crystal structure and other spectroscopic data matched the previously published data (Figure S32).^7b,21^ Since THF coordinates stronger to the scandium center than triethylamine, using THF exclusively for the tetramethylaluminato cleavage gave the previously reported Cp*_2_ScMe­(thf) (**3^thf^**).^7b^ Because to date only ^1^H NMR data of **3^thf^** seemed available, its comprehensive analysis was performed by ^1^H, ^13^C­{^1^H}, and ^45^Sc NMR spectroscopy in C_6_D_6_, SC-XRD as well as elemental analysis. As expected, donor (THF) coordination in **3** ^**thf**^ causes a significant ^45^Sc chemical shift to higher field (δ = 151.5 versus 220 ppm).^21^ The Ln–C­(methyl) distance in **3** ^**thf**^ (2.2748(15) Å) is shorter than in the larger metal congeners Cp*_2_LnMe­(thf) (Ln = Y: 2.44(2) Å, Sm: 2.484(14) Å, Lu: 2.33(1) Å)^10,22^ but longer than in donor-free **3** (2.266(3) Å). These findings are consistent with the different rare-earth-metal ion sizes and decreased coordination number, respectively. As known for the congener Cp*_2_Y­(AlMe_4_), donor-induced aluminate cleavage of Cp*_2_Ln­(AlMe_4_) (Ln = Sc, Y) with THF can be reversed *in vacuo*.^23^

### Cp*_2_Sc­(GaMe_4_), Cp*_2_Sc­(InMe_4_), and Methylidene Clusters Cp*_6_Sc_4_E_8_(CH_2_)_12_Me_6_ (E = Ga, In)

Since homoleptic Sc­(AlMe_4_)_3_ was reported as a temperature-sensitive compound, it could be envisaged that Sc­(GaMe_4_)_3_ would be even more sensitive. Attempts to obtain Sc­(GaMe_4_)_3_ from [ScMe_3_]_*n*_ with excess GaMe_3_ at low temperatures did not indicate a reaction at all. Therefore, the donor-assisted tetramethylaluminate/tetramethylgallate exchange protocol introduced in 2010 was considered.^15^ There, it was revealed that a donor-assisted exchange of AlMe_3_ in La­(AlMe_4_)_3_ (“LaMe_3_×3AlMe_3_”) for the less Lewis acidic GaMe_3_ proceeded using diethyl ether and excess GaMe_3_, followed by crystallization. More recently, this exchange protocol was also successfully applied for converting Ln­(AlMe_4_)_3_ into the first rare-earth-metal tetramethylindates.^24^ Here, according to this approach, scandocene aluminate **1-Al** was treated with slight excess of EMe_3_ (E = Ga, In) and diethyl ether. The corresponding gallium and indium compounds Cp*_2_Sc­(GaMe_4_) (**1-Ga**) and Cp*_2_Sc­(InMe_4_) (**1-In**), respectively could be obtained in moderate crystalline yields (Scheme 2).

**Scheme 2:**
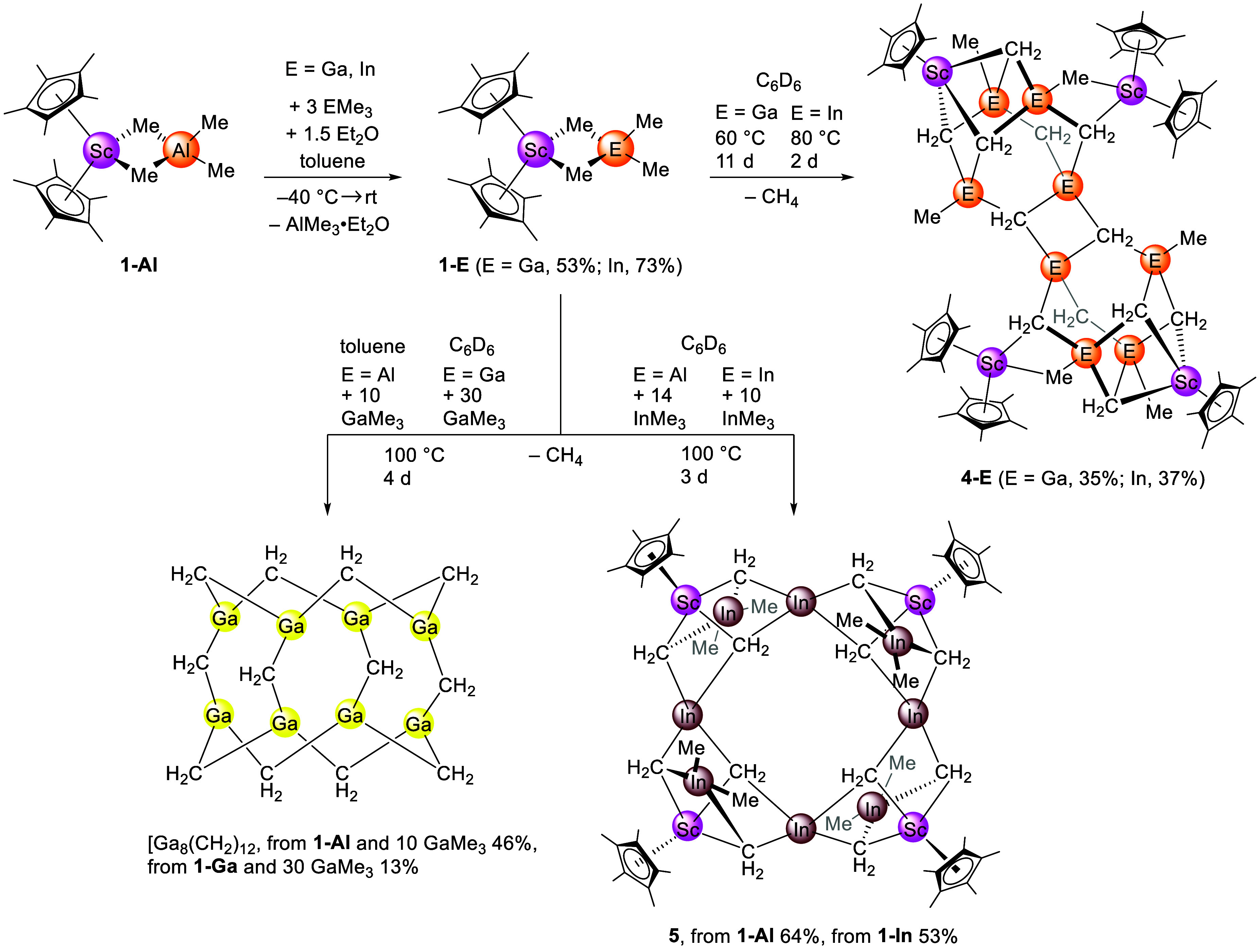
Synthesis of Cp*_2_Sc­(GaMe_4_) (**1-Ga**), Cp*_2_Sc­(InMe_4_) (**1-In**), Methylidene Clusters Cp*_6_Sc_4_E_8_(CH_2_)_12_Me_6_ (**4-E**, E = Ga, In) and Cp*_4_Sc_4_In_8_(CH_2_)_12_Me_8_ (**5**), as well as Known Ga_8_(CH_2_)_12_

Tetramethylgallate **1-Ga** crystallized isostructural to **1-Al** as a monomer (Figure 1/top). The Sc–C­(methyl) distances in **1-Ga** are slightly longer than in **1-Al** (2.5072(16) Å and 2.5196(17) Å for **1-Ga** versus 2.490(5) Å and 2.505(5) Å for **1-Al**). Tetramethylindate **1-In**, on the other hand, crystallizes as a dimer with one cocrystallized molecule of InMe_3_ (Figure 1/bottom).

**Figure 1:**
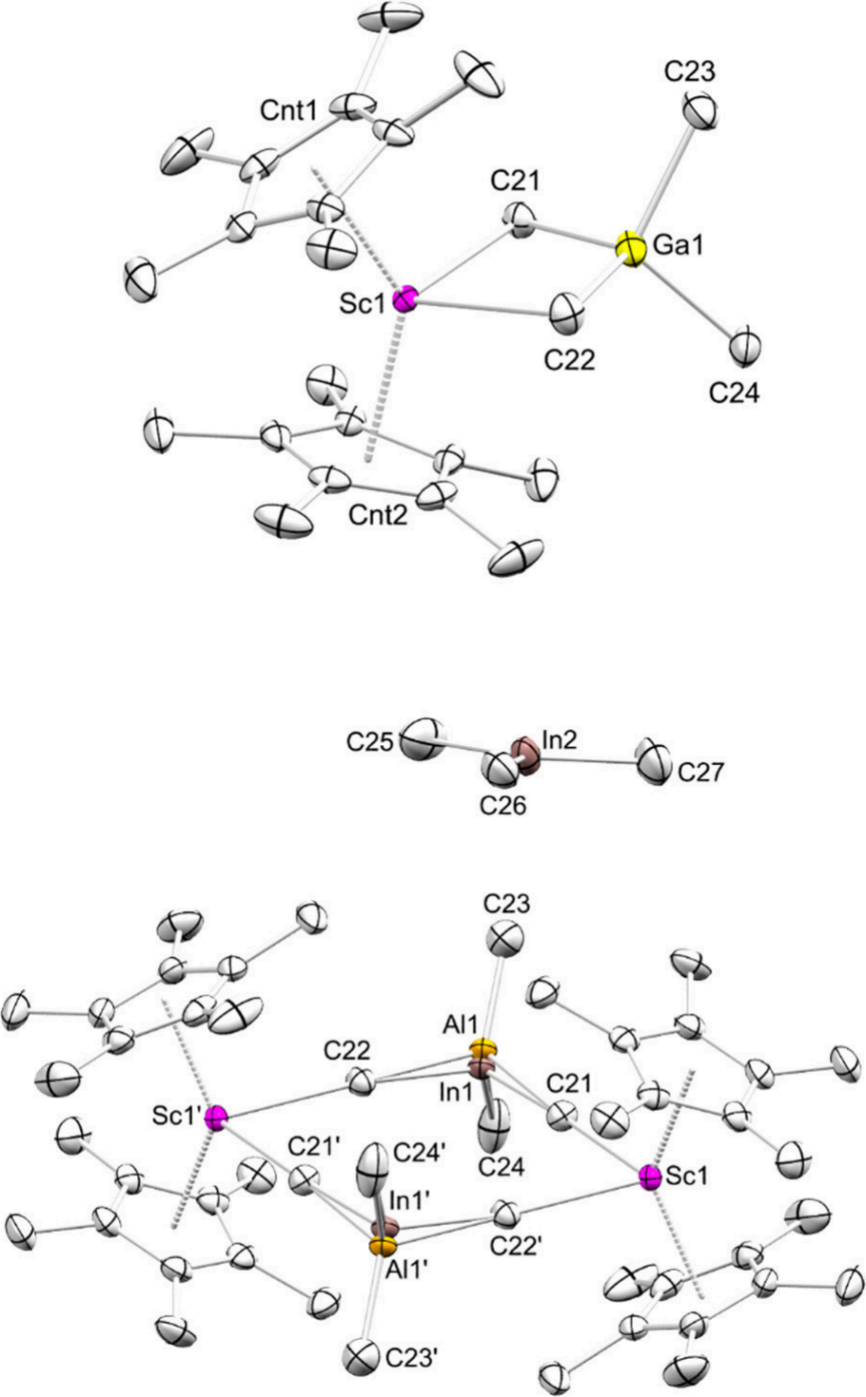
Crystal structures of **1-Ga** (top) and **1-In** (bottom). Atomic displacement ellipsoids were set at 50% probability. Hydrogen atoms omitted for clarity. For **1-Ga**, a second molecule of Cp*_2_Sc­(GaMe_4_) was omitted for clarity. For **1-In**, a molecule of toluene was omitted for clarity. Selected interatomic distances [Å] and angles [deg]: **1-Ga**: Sc1–C21 2.5072(16), Sc1–C22 2.5196(17), Ga1–C21 2.0957(16), Ga1–C22 2.0935(18), Sc1···Ga1 2.9470(3), C21–Sc1–C22 88.46(6), Sc1–C21–Ga1 79.06(5). **1-In**: Sc1–C21 2.561(2), Sc1–C22′ 2.592(3), In1–C21 2.154(2), In1–C22 2.140(2), Al1–C21 2.215(4), Al1–C22 2.194(4), C21–Sc1–C22′ 83.24(7), Sc1–C21–In1 176.32(11), Sc1–C21–Al1 173.33(14) (see also Supporting Information Figure S35 and S36).

At this point, it is important to mention that the exchange reactions are not complete. As expected for ion exchange reactions, there is a definite amount of residual aluminum left in **1-Ga** and **1-In**. Examining different excesses of EMe_3_ resulted in similar incomplete transformations. The ratios of Ga:Al and In:Al were determined via ICP-OES as 2.75:1 and 1.94:1, respectively. This is also seen in the ^1^H and ^45^Sc NMR spectra showing signals of Cp*_2_Sc­(AlMe_4_) besides **1-Ga** and **1-In**, respectively (Figure 2). Repeating the reaction of preisolated aluminum-containing Cp*_2_ScGaMe_4_ (**1-Ga**) with GaMe_3_ and Et_2_O did not substantially improve the Ga:Al ratio, based on ^1^H and ^45^Sc NMR spectra. The signals of Cp*_2_Sc­(AlMe_4_), Cp*_2_Sc­(GaMe_4_), and Cp*_2_Sc­(InMe_4_) separate well in the variable temperature ^45^Sc NMR spectra (Figure S13 and S14 for **1-Ga**, Figure S20 and S21 for **1-In**). The cocrystallized InMe_3_ in **1-In** can be removed via prolonged evacuation, since InMe_3_ is a volatile solid at ambient temperature (bp. 136 °C).^25^ The loss of InMe_3_ is indicated by the ^1^H and ^45^Sc NMR spectra (Figure 2), elemental analysis, and weight loss. In contrast to the recently reported homoleptic rare-earth-metal tetramethylindates Ln­(InMe_4_)_3_ (Ln = La, Ce, Nd), sandwich complex **1-In** is stable at ambient temperature.^24^ Like **1-Al**, **1-Ga** and **1-In** show monomer dimer equilibria in variable temperature ^1^H NMR studies (Figure S13 and S20).

**Figure 2:**
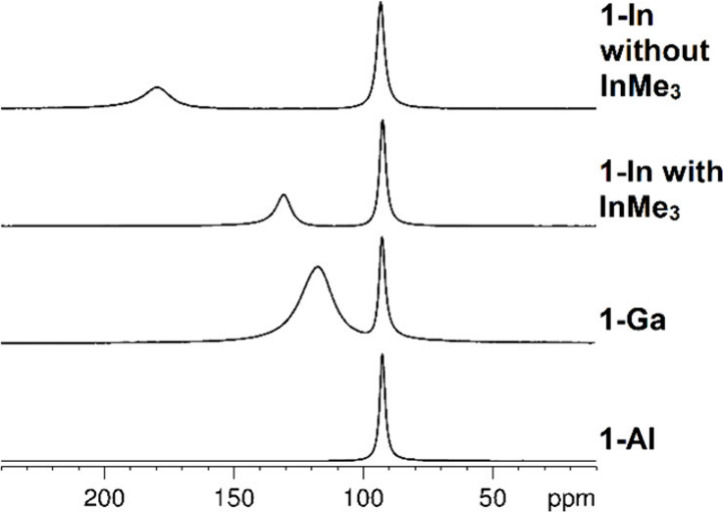
^45^Sc NMR spectra of **1-Al**, **1-Ga**, **1-In** with cocrystallized InMe_3_, and **1-In** without cocrystallized InMe_3_.

We have previously found that the yttrium congeners Cp*_2_Y­(EMe_4_) (E = Al, Ga) activate the benzene C–H bond above 100 °C.^9^ Accordingly, deprotonation of C_6_D_6_ by a CH_3_ ligand evolves CDH_3_, which was identified by its ^1^H NMR triplet signature. When heating C_6_D_6_ solutions of scandium complexes **1-E** (E = Al, Ga, In) in J. Young-valved NMR tubes instead, almost exclusive elimination of CH_4_ was detected. Thus, C–H-bond activation of a methyl group in **1-E** is favored over the intermolecular activation of C_6_D_6_. This might be due to scandium being the smallest rare-earth-metal ion and, in comparison with the larger rare-earth metals, leaving less space for additional close coordination of aromatic solvent molecules. For **1-Al**, after 6 days at 100 °C, a yellow oil was obtained, which contained a mixture of ill-defined products. For **1-Ga** and **1-In**, methane evolution occurred at 60 and 80 °C, respectively. Same as for **1-Al**, CH_4_ evolution was favored over CDH_3_ evolution. In these reactions, after 11 days at 60 °C for **1-Ga**, and after 2 days at 80 °C for **1-In**, crystalline solids were forming in the NMR tube. Upon separation from the supernatants, the crystalline solids were analyzed by SC-XRD as dodecametallic clusters Cp*_6_Sc_4_E_8_(CH_2_)_12_Me_6_ (**4-E**) (E = Ga, In). The core of compounds **4-E** consists of two fused adamantane-like methyl triel methylidene moieties (Scheme 2, Figure 3).

**Figure 3:**
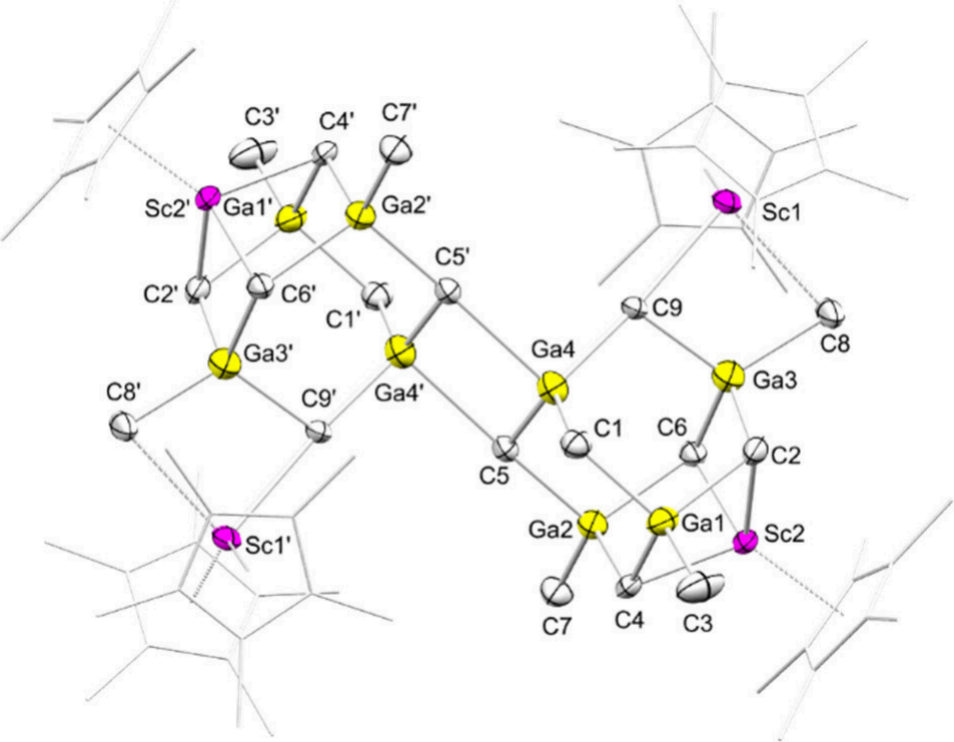
Connectivity crystal structure of **4-Ga**. Atomic displacement ellipsoids were set at 50% probability. Hydrogen atoms omitted for clarity. Two molecules of toluene were omitted for clarity. The Cp* rings are shown in wireframe for clarity.

Each E_4_(CH_2_)_6_Me_3_ (E = Ga, In) moiety in complexes **4-E** is capped with one sandwich and one half-sandwich scandium fragment. While the Cp*_2_Sc fragment is linked to one methylidene, and over a longer distance to one methyl group, the mono-Cp* scandium moiety is additionally coordinated by three methylidenes in a piano-stool fashion. Apparently, in the process of the formation of **4-E** from **1-E** (E = Ga, In), Cp* ligands get displaced. As for precursors **1-E** (E = Ga, In), **4-E** contained residual aluminum (ICP-OES: **4-Ga**, Ga:Al of 4.53:1; **4-In**, In:Al of 1.74:1).

### Known Ga_8_(CH_2_)_12_ and New Cp*_4_Sc_4_In_8_(CH_2_)_12_Me_8_ , as well as Me_2_InCp*InMe_3_

^10^ Next, compounds **1-E** (E = Al, Ga, In) were heated in the presence of additional EMe_3_. The reaction of **1-Al** with 100 equiv AlMe_3_ at 120 °C yielded a yellowish precipitate. A ^1^H NMR spectrum of the product in THF-*d*_8_ showed signals between −0.96 and −1.81 ppm, similar to those reported for methyl aluminum methylidene [MeAl­(CH_2_)]_12_.^11^ But the product derived from scandocene **1-Al** and excess AlMe_3_ contained a Cp*Sc species that could not be removed during washing steps with different solvents. Minor signals in the Cp* region in the ^1^H NMR spectrum and a peak in the ^45^Sc NMR spectrum at 139.7 ppm remained (Figure S27 and S28).

Aiming at known gallium methylidene Ga_8_(CH_2_)_12_, we then performed reactions of **1-Ga** with GaMe_3_. It was previously described that Ga_8_(CH_2_)_12_ can be obtained when excess GaMe_3_ is thermally treated at 130 °C in the presence of lutetium congener Cp*_2_Lu­(GaMe_4_).^10^ Consistently, we found that Ga_8_(CH_2_)_12_ is formed in the reaction of **1-Ga** with 30 equiv GaMe_3_ (Scheme 2). Crucially, when skipping the aluminate/gallate exchange reaction and hence the isolation of **1-Ga**, treatment of **1-Al** with 10 equiv GaMe_3_ did also afford Ga_8_(CH_2_)_12_. ICP-OES analysis confirmed that Ga_8_(CH_2_)_12_ obtained from **1-Al** contained a Ga:Al ratio of 8.16:1.

With this reactivity on hand, we tackled the synthesis of elusive indium methylidene. However, the reaction of **1-In** with 10 equiv InMe_3_ at 100 °C for 3 days in C_6_D_6_ did not yield a homoleptic indium methylidene. Instead, the crystalline solid formed during the reaction was identified as Cp*_4_Sc_4_In_8_(CH_2_)_12_Me_8_ (**5**) via SC-XRD (Figure 4). Other than for the gallium case, scandium atoms remained in the complex even when trying different temperatures or different equivalents of InMe_3_. Dodecametallic cluster **5** was obtained as well in a reaction of **1-Al** with 14 equiv InMe_3_ over 3 days at 100 °C. ICP-OES revealed that **5** obtained from **1-Al** features a In:Al ratio of 10.07:1. The structure of **5** consists of a tetrameric half-sandwich scandium indium methylidene methyl cluster. Four indium atoms are each surrounded by four methylidene groups and are located in the plane spanned by the four scandium atoms. The other four indium atoms are each surrounded by two methylidenes and two terminal methyl groups and are located alternately below and above the Sc_4_ plane. As in **4-E** (E = Ga, In), the half-sandwich scandium moieties each are linked to three methylidene groups in a piano-stool fashion. Overall, compound **5** can be described as a methyl indium methylidene stabilized scandium methylidene, {[Cp*ScCH_2_]­(Me_2_In_2_(CH_2_)_2_)}.

**Figure 4:**
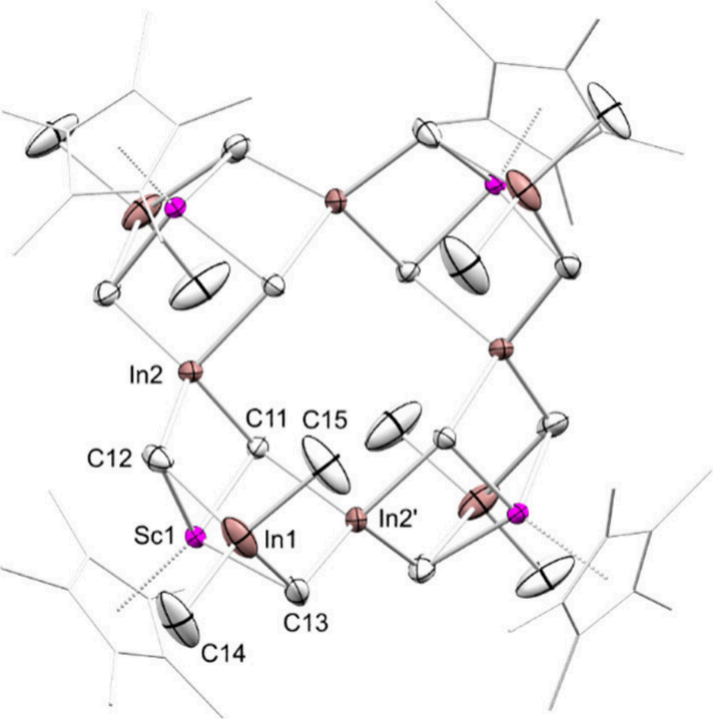
Crystal structure of **5**. Atomic displacement ellipsoids were set at 50% probability. Hydrogen atoms omitted for clarity. The Cp* rings are shown in wireframe for clarity. Selected interatomic distances [Å] and angles [deg]: Sc1–C11 2.306(7), Sc1–C12 2.255(8), Sc1–C13 2.265(8), In1–C12 2.346(10), In1–C13 2.340(9), In1–C14 2.184(9), In1–C15 2.174(11), In2–C11 2.275(7), In2–C12 2.250(8), In2’–C11 2.266(7), In2’–C13 2.254(8), C11–Sc1–C12 98.5(3), C11–Sc1–C13 97.2(3), C12–Sc1–C13 100.0(3) (see also Supporting Information Figure S39).

To the best of our knowledge, this is the third report on structurally characterized indium methylidenes. In 1986, compounds Cl_2_In­(tmeda)­CH_2_In­(tmeda)­Cl_2_ and ClBrIn­(tmeda)­CH_2_In­(tmeda)­Cl_2_ (tmeda = N,N,N′,N′-tetramethylethylenediamine) were obtained from a reaction of indium monohalides, indium trichloride and dichloromethane.^26^ More recently, ancillary ligand free undecametallic cluster La_4_In_7_(C)­(CH)_2_(CH_2_)_2_(Me)_19_ was isolated from thermal decomposition of homoleptic La­(InMe_4_)_3_.^24^

Crystals of **4-Ga** were of low quality and yielded only a connectivity crystal structure. Therefore, the structural properties of **4-In** and **5**, but not **4-Ga**, will be discussed. The Sc–C­(methylidene) distances in the [Cp*Sc­(CH_2_)_3_]^4–^ fragments of **4-In** (2.224(5)–2.456(5) Å) are slightly shorter than those in **5** (2.255(8)–2.306(7) Å). Both are comparable to other multinuclear scandium methylidene compounds, comprising [Cp*Sc­(μ_3_–CH_2_)]_4_ (2.210(8)–2.303(7) Å), Sc_3_(μ_4_–CH_2_)_2_(μ_2_–Me)_3_(AlMe_4_)_2_(AlMe_3_)_2_ (2.298(3)–2.442(2) Å), {(NCN)­Sc­(μ_2_–Me)}_3_(μ_3_–Me)­(μ_3_–CH_2_) (NCN = [PhC­{NC_6_H_4_(*i*Pr-2,6)_2_}_2_]^−^) (avg. 2.367(4) Å), (LSc)_2_(μ–CH_2_)­(μ–PDipp) (L = [MeC­(NDipp)­CHC­(NDipp)­Me]^−^, Dipp = 2,6-(*i*Pr)_2_C_6_H_3_) (2.193(3)–2.232(3) Å).^5a,12,27^ The Sc–C­(methylidene) distance in the sandwich [Cp*_2_Sc­(CH_2_)­Me]^2–^ fragment of **4-In** is much larger (2.445(4) Å), while the Sc–C­(methyl) distance in the [Cp*_2_Sc­(CH_2_)­Me]^2–^ fragment is even larger (2.574(5) Å). In **4-In** and **5**, all indium atoms are tetracoordinate and all methylidene groups are μ_3_–CH_2_. The In–C­(methylidene) distances in **4-In** (1.990(7)–2.489(5) Å) differ significantly from each other due to the constrained geometry of two fused adamantane-like cluster cores. The In–C­(methylidene) distances in **5** (2.250(8)–2.346(10) Å) are similar to the In–C­(methylidene) distance in La_4_In_7_(C)­(CH)_2_(CH_2_)_2_(Me)_19_ (2.277(4) Å),^24^ but are significantly longer than the Ga–C­(methylidene) distances in Ga_8_(CH_2_)_12_ (1.960(2)–1.972(2) Å).^10^ Part of this difference may reflect the distinct coordination numbers of gallium in Ga_8_(CH_2_)_12_ (CN = 3) and indium in **5** (CN = 4) However, the tetracoordinate gallium atoms in Cp*_6_Lu_3_(μ_3_–CH_2_)_6_Ga_9_(μ–CH_2_)_9_ exhibit Ga–C­(methylidene) distances for μ_3_–CH_2_ groups of 2.125(6) Å.^10^ Furthermore, the In–C­(methylidene) distances in **4-In** and **5** are significantly longer than that detected for the pentacoordinate indium atoms and the μ_2_–CH_2_ group in Cl_2_In­(tmeda)­CH_2_In­(tmeda)­Cl_2_ (2.147(4) Å).^26^ Also in **5**, the In–C­(methylidene) distances are greater in average than the Sc–C­(methylidene) distances.

Complexes **1-In**, **4-In**, and **5** are the first examples of complexes containing scandium and indium. Hence, these are the first examples of organometallic rare-earth-metal complexes with a trivalent group 13 metal in which the group 13 metal has a larger ionic radius than the rare-earth metal. The effective ionic radii of hexacoordinate trivalent scandium and indium are 0.745 Å and 0.800 Å, respectively.^13^ This results in a size mismatch, which often involves low stability at ambient temperature, especially for homoleptic complexes Sc­(AlMe_4_)_3_ or La­(InMe_4_)_3_.^12,24,28^ La­(GaMe_4_)_3_ and La­(AlMe_4_)_3_ with matching sizes, on the other hand, are stable at ambient temperature.^15^ Therefore, the [Cp*_2_Sc]^+^ sandwich moiety is required to stabilize the [GaMe_4_]^−^ and [InMe_4_]^−^ units kinetically.

The different products obtained from **1-Al** with GaMe_3_ and InMe_3_, respectively, are striking. Homoleptic Ga_8_(CH_2_)_12_ is formed readily in moderate yields from **1-Al** and 10 equiv GaMe_3_. But in the indium case, the [Cp*Sc]^2+^ fragment sticks to methyl indium methylidene. **4-E** (E = Ga, In) and **5** could not be analyzed via NMR spectroscopy, as they are neither soluble in aromatic hydrocarbon solvents nor 1,2-difluorobenzene. The synthesis of **5** was monitored via NMR spectroscopy to yield a silent ^45^Sc NMR spectrum at the end (Figure S29). When dissolved in THF cluster disassembly prevailed. The ^1^H and ^45^Sc NMR spectra of **4-E** (E = Ga, In) and **5** in THF-*d*_8_ showed signals of Cp*_2_ScMe­(thf) (**3** ^**thf**^) as the main degradation product (Figure S22 and S23).

Curiously enough, the formations of **4-E** (E = Ga, In) and **5** proceed via displacement of Cp* ligands at the scandium centers. The scandocene precursors are partly (**4-E**) or fully (**5**) converted into half-sandwich scandium entities. Such half-sandwich formation is in contrast to the methylidene complexes Cp*_6_Ln_3_(CH_2_)_15_Ga_9_ (Ln = Y, Lu) obtained from Cp*_2_Ln­(GaMe_4_) and two equivalents GaMe_3_ in methylcyclohexane, or Cp*_4_Lu_2_(CH_2_)_12_Al_10_Me_8_ obtained from Cp*_2_Lu­(AlMe_4_) and four equivalents AlMe_3_ in toluene-*d*_8_.^10,11^ In these methylidene complexes, the [Cp*_2_Ln]^+^ fragments are still intact. Thus, compounds **4-E** (E = Ga, In) and **5** may present additional insights into the formation of Ga_8_(CH_2_)_12_ from Cp*_2_Ln­(GaMe_4_) (Ln = Sc, Y, Lu). We found that **4-Ga** does react with additional GaMe_3_ at elevated temperatures to form Ga_8_(CH_2_)_12_. The displacement of Cp* ligands has been reported earlier, mainly when Cp*-supported compounds were reacted with KC_8_. A recent example from Evans and co-workers described the formation of a mixed sandwich half-sandwich scandium dinitrogen compound in a reaction of a reduced sandwich scandium dinitrogen complex with KC_8_.^17^ There, the reduction with KC_8_ triggered the precipitation of KCp*. Additionally, they reported on a half-sandwich scandium oxo hydroxo cluster formed from the sandwich compound (Cp*_2_Sc)_2_(N_2_) with water impurities.

A plausible reaction pathway for the formation of half-sandwich entities in complexes **4-E** (E = Ga, In) and **5** is a preferred Sc → group 13 cyclopentadienyl transfer. To support this hypothesis, InMe_3_ was reacted with HCp* in C_6_D_6_ for 12 days at 110 °C. After this time period, there were still signals of the HCp* proligand visible in ^1^H NMR spectra. However, removal of volatiles, extracting the remaining solid with *n*-hexane, and cooling the extract to −40 °C for 1 day yielded colorless single crystals of Me_2_InCp*InMe_3_ (**6**). The [Me_2_In]^+^ fragment of **6** is coordinated by the Cp* ligand in an η^2^ fashion with distinct In–C­(Cp*) distances of 2.407(2) Å and 2.561(2) Å (Figure 5). On the opposite side, the Cp* ligand connects to the InMe_3_ moiety in an η^1^ fashion with an In–C­(Cp*) distance of 2.590(2) Å. Such inverse sandwich-type coordination seems unprecedented in trivalent group 13 Cp* complexes but is known for monovalent derivatives.^29^ The ^1^H NMR spectrum of **6** in C_6_D_6_ shows one signal for the Cp* protons, while the methyl groups bound to indium appeared as one slightly broadened signal. Solid **6** turned yellow upon prolonged evacuation, and the ^1^H NMR spectra indicated loss of InMe_3_. Thus, no elemental analysis was performed. The supernatants of the reaction mixtures of **4-E** and **5** contained multiple signals of Cp* as well as metal-bonded methyls in the ^1^H NMR spectra and multiple signals in the ^45^Sc NMR spectra. For **4-In**, there were multiple Cp* signals including a small one at 1.99 ppm. For **5**, the by far most intense Cp* signal is located at 1.98 ppm, which matches the Cp* shift measured for **6** (δ 1.99 ppm). Therefore, it can be assumed that especially in the synthesis of **5**, with an excess of InMe_3_ being present throughout, a Sc → group 13 cyclopentadienyl transfer is likely to form **6** or a similar compound. However, attempts to isolate **6** from the reaction mixture of **5** were not successful.

**Figure 5:**
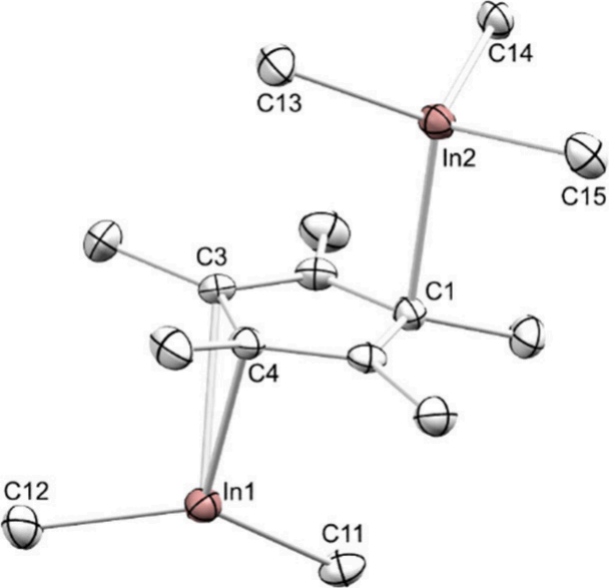
Crystal structure of **6**. Atomic displacement ellipsoids were set at 50% probability. Hydrogen atoms and a second molecule of **6** omitted for clarity. Selected interatomic distances [Å] and angles [deg]: In1–C3 2.561(2), In1–C4 2.407(2), In1–C11 2.142(2), In1–C12 2.154(2), In2–C1 2.590(2), In2–C13 2.164(2), In2–C14 2.235(2), In2–C15 2.180(2), C11–In1–C12 137.74(10) (see also Supporting Information Figure S40).

In search of putative homoleptic indium methylidene, we also attempted analog reactions which were successful for the synthesis of methyl aluminum methylidene [MeAl­(CH_2_)]_12_.^11^ Accordingly, Ga_8_(CH_2_)_12_ reacts with AlMe_3_ to form [MeAl­(CH_2_)]_12_. Treatment of Ga_8_(CH_2_)_12_ with InMe_3_, however, showed no reaction even at 80 °C. Cp_2_TiCl_2_ catalyzes the reaction of 100 equiv AlMe_3_ to [MeAl­(CH_2_)]_12_ at ambient temperature.^30^ A reaction of Cp_2_TiCl_2_ with excess InMe_3_ yielded crystals identified as Me_2_InCl by a unit cell check.^31^ The reaction of Cp_2_TiCl_2_ with 50 equiv InMe_3_ at 100 °C yielded gray solid blocks. This solid did not even dissolve in water, only in half-concentrated hydrochloric acid. We therefore assume that the precipitate is indium metal. InMe_3_ is known to decompose and form indium metal at high temperatures.^32^ Therefore, very high temperatures are not productive for the synthesis of elusive homoleptic indium methylidene.

As mentioned above, methylidenes **4-E** (E = Ga, In) and **5** are neither soluble in aromatic hydrocarbon solvents nor 1,2-difluorobenzene. Decomposition was observed when dissolved in THF. Therefore, a methylenation reaction was attempted in a J. Young-valved NMR tube, employing a suspension of 10.1 mg of **5** and 6 equiv. fluorenone in C_6_D_6_. Over the course of 3 days at ambient temperature, the amount of fluorenone decreased, while the amount of methylenation product dibenzofulvene increased, as evidenced by the ^1^H NMR spectra (Figure S30). The ^45^Sc NMR spectrum remained silent.

## Conclusions

Monomeric and dimeric heterobimetallic complexes Cp*_2_Sc­(AlMe_4_), Cp*_2_ScMe­(thf), [Cp*_2_Sc­(ClAlMe_3_)]_2_ can be conveniently synthesized. Donor-assisted trimethyltriel exchange in Cp*_2_Sc­(AlMe_4_) gives access to Cp*_2_Sc­(EMe_4_) (E = Ga, In), representing the first scandium tetramethylgallate and tetramethylindate complexes. While Cp*_2_Sc­(GaMe_4_) is isostructural to Cp*_2_Sc­(AlMe_4_), the indium congener forms a dimer and cocrystallizes with InMe_3_. When heated, solutions of **1-E** (E = Al, Ga, In) in deuterated solvents such as C_6_D_6_ or toluene-*d*_8_ eliminate CH_4_, instead of CDH_3_, generating mixed methylidene/methyl clusters Cp*_6_Sc_4_E_8_(CH_2_)_12_Me_6_ (E = Ga, In). This suggests that unlike the congeners of the larger rare-earth metals, scandocenes Cp*_2_Sc­(EMe_4_) (E = Al, Ga, In) prefer activation of internal methyl groups over activation of external aromatic solvents. Heating of Cp*_2_Sc­(EMe_4_) (E = Al, Ga) in the presence of excess GaMe_3_ gave the previously reported homoleptic Ga_8_(CH_2_)_12_. In contrast, performing the reaction of Cp*_2_Sc­(EMe_4_) (E = Al, In) with an excess of InMe_3_ led to the isolation of Cp*_4_Sc_4_In_8_(CH_2_)_12_Me_8_, instead of putative homoleptic indium methylidene. This study shows that heterobimetallic methyl complexes with anticipated size mismatches such as Sc/Ga and Sc/In can be obtained with proper kinetic stabilization such as a scaffold of Cp* ligands. The study also revealed that rare-earth metal promoted reactions that led to the isolation of Ga_8_(CH_2_)_12_ and [MeAl­(CH_2_)]_12_, proceed differently for the group 13 metal indium.

## Experimental Section

### General Considerations

***Caution!*** *LiAlMe*_*4*_ *, AlMe*_*3*_ *, GaMe*_*3*_ *, InMe*_*3*_ *and their derivatives are highly pyrophoric and react violently when exposed to air and/or moisture*. All manipulations were performed under an inert argon atmosphere, either by using Schlenk techniques or a glovebox (MBraun 200B; <0.1 ppm of O_2_, <0.1 ppm of H_2_O). *n*-Hexane, *n*-pentane, tetrahydrofuran (THF), diethyl ether (Et_2_O), and toluene were dried over Grubbs columns (MBraun SPS, solvent purification system) and stored over 3 Å molecular sieves inside a glovebox. C_6_D_6_, THF-*d*_8_, and toluene-*d*_8_ were purchased from Sigma-Aldrich, degassed, stirred over NaK for at least 24 h, filtered and stored inside a glovebox. Sc_2_O_3_ was purchased from abcr. AlMe_3_ and InMe_3_ were purchased from abcr and used as received. GaMe_3_ was purchased from Dockweiler Chemicals and used as received. *n*-BuLi was purchased from Sigma-Aldrich. HC_5_Me_5_ (HCp*) was purchased from BLDPharm. MeLi was purchased from Sigma-Aldrich and the solvent was removed before storing inside a glovebox. ScCl_3_(thf)_3_,^33^ LiCp*^34^ (Cp* = C_5_Me_5_), and LiAlMe_4_ (ref 35) were synthesized according to literature procedures. ^1^H, ^13^C­{^1^H} and ^45^Sc NMR spectra were recorded on a Bruker Avance AVII+400, a Bruker Avance III HD 300 NanoBay or a Bruker AVII+500. ^1^H, ^13^C, and ^45^Sc NMR chemical shifts are referenced to internal solvent resonances and reported in parts per million (ppm) relative to tetramethylsilane (TMS), and Sc­(NO_3_)_3_. Analysis of NMR spectra were performed with Bruker Topspin (Version 3.6.1). Multiplicities of signals are given as s (singlet), m (multiplet) and br (broad). Crystals for X-ray crystallography were handpicked in a glovebox, coated with Parabar 10312 and stored on microscopic slides. Crystallographic data collection was done on a Bruker APEX II Duo diffractometer using QUAZAR optics for Mo K_α_ (λ = 0.71073 Å). The data collection strategy was determined using COSMO^36^ with ω and φ scans. Raw data were processed by APEX^37^ and SAINT,^38^ corrections for absorption effects were applied using SADABS.^39^ The structures were solved by direct methods and refined against all data by full-matrix least-squares methods on F^2^ using SHELXTL^40^ and Shelxle.^41^ Disorder models were calculated using DSR,^42^ a program included in Shelxle for refining disorder. Structure plots were generated by using MERCURY^43^ and POV-Ray.^44^ Elemental analysis (C, H, N) was performed on an Elementar vario MICRO cube. Aluminum and indium contents were determined via ICP-OES on a Thermo Scientific iCAP 7000 Series.

### Cp*_2_Sc­(AlMe_4_) (1-Al)

Solid LiCp* (752.0 mg, 5.289 mmol) was added to a stirred suspension of ScCl_3_(thf)_3_ (918.3 mg, 2.498 mmol) and AlMe_3_ (357 mg, 4.96 mmol) in 5 mL toluene. The resulting pale-yellow suspension was stirred for 3 days at 120 °C. The mixture was filtered, and the remaining solution was added to a cooled, stirred suspension of LiAlMe_4_ (235 mg, 2.49 mmol) and AlMe_3_ (550 mg, 7.63 mmol) in toluene. The resulting yellow suspension was stirred at ambient temperature overnight. The mixture was filtered, and the solvent was removed *in vacuo*. The resulting solid was recrystallized from a toluene/*n*-hexane mixture at −40 °C to yield 724 mg (1.79 mmol, 72%) of **1-Al** as colorless crystals. ^1^H and ^45^Sc NMR spectra were in line with previously published data.^12^

### [Cp*_2_ScClAlMe_3_]_2_ (2)

Solid LiCp* (260.9 mg, 1.835 mmol) was added to a stirred suspension of ScCl_3_(thf)_3_ (306.7 mg, 0.834 mmol) and AlMe_3_ (119 mg, 1.65 mmol) in 4 mL toluene. The resulting pale-yellow suspension was stirred for 3 days at 120 °C. The mixture was filtered, and the remaining solution was cooled to −40 °C. Neat AlMe_3_ (180 mg, 2.50 mmol) was added to the cooled, stirred solution and the resulting yellow suspension was stirred overnight at ambient temperature. The mixture was filtered, and the solvent of the supernatant was removed *in vacuo*. The resulting solid was recrystallized from toluene at −40 °C to yield 274 mg (0.324 mmol, 78%) of **2** as yellow crystals. ^1^H NMR (500 MHz, toluene-*d*_8_, 26 °C): δ 1.80 (s, 30H, C_5_ Me_5_), 1.76 (s, 30H, C_5_ Me_5_), −0.11 (s, 9H, AlMe_3_), −0.34 (br, 9H, AlMe_3_), −0.60 (br, 9H, AlMe_3_) ppm. ^13^C­{^1^H} NMR (126 MHz, toluene-*d*_8_, 26 °C): δ 123.8 (C_5_Me_5_), 120.4 (C_5_Me_5_), 12.1 (C_5_ Me_5_), 11.7 (C_5_ Me_5_) ppm. The ^13^C­{^1^H} NMR signal of the methyl group bound to scandium and aluminum could not be resolved. ^45^Sc NMR (122 MHz, toluene-*d*_8_, 26 °C): δ 167.7, 123.5, 92.8 ppm. C_46_H_78_Al_2_Cl_2_Sc_2_ (845.90 g/mol): calcd. (%) C 65.32, H 9.29; found C 67.40, H 9.38. Although these results are outside the range viewed as establishing analytical purity (C: +2.08%), they are provided to illustrate the best values obtained to date. The increased carbon contents may likely result from toluene in the crystalline lattice.

### Cp*_2_ScMe (3)

A suspension of **1-Al** (76.0 mg, 0.189 mmol) in 2 mL *n*-hexane was precooled to −40 °C. Et_2_O (0.3 mL) was added to the cooled suspension at −40 °C. The mixture became clear after 5 min at −40 °C. After 1 day at −40 °C, Et_3_N (0.5 mL) was added at −40 °C. After 1 day at −40 °C, the slightly yellow solution was concentrated to about 1 mL *in vacuo*. After 3 days at −40 °C, yellow crystals of AlMe_3_(NEt_3_) formed. The supernatant was collected and volatiles were removed *in vacuo*. The remaining solid was dissolved in *n*-pentane and cooled to −40 °C. After 5 days, colorless crystals formed, and the supernatant was removed to yield **3** (33.1 mg, 0.100 mmol, 53%). ^1^H and ^45^Sc NMR spectra were in line with previously published data.^7b,21^

### Cp*_2_ScMe­(thf) (3^thf^)

A suspension of **1-Al** (154.1 mg, 0.383 mmol) in 3 mL *n*-hexane was precooled to −40 °C. THF (3 mL) was added to the cooled suspension at ambient temperature. The mixture became clear after 5 min at ambient temperature. After 1 week at −40 °C, huge colorless crystals formed. The supernatant was removed and the crystals were washed with 0.5 mL cold *n*-hexane to yield **3** (122 mg, 0.303 mmol, 79%). ^1^H NMR (400 MHz, C_6_D_6_, 26 °C): δ 3.47 (m, 4H, α-THF), 1.94 (s, 30H, C_5_ Me_5_), 1.35 (m, 4H, β-THF), −0.37 (s, 3H, ScMe) ppm. ^13^C­{^1^H} NMR (101 MHz, C_6_D_6_, 26 °C): δ 117.4 (C_5_Me_5_), 68.7 (α-THF), 25.6 (β-THF), 12.0 (C_5_ Me_5_) ppm. The ^13^C­{^1^H} NMR signal of the methyl group bound to scandium could not be resolved. ^45^Sc­{^1^H} NMR (97 MHz, C_6_D_6_, 26 °C): δ 151.5 ppm. C_25_H_41_OSc (402.56 g/mol): calcd. (%) C 74.59, H 10.27; found C 75.04, H 10.08.

### Cp*_2_Sc**(G**aMe_4_) (1-Ga)

A solution of GaMe_3_ (198 mg, 1.72 mmol) and Et_2_O (60 mg, 0.81 mmol) in 2 mL toluene was added at −40 °C to a cooled, stirred solution of **1-Al** (210 mg, 0.522 mmol) in 2 mL toluene. The mixture became slightly cloudy but went back to clear after 5 min during warming to ambient temperature. The resulting yellow solution was concentrated to 2 mL and cooled to −40 °C for crystallization. After 10 days at −40 °C, slightly yellow crystals formed. The supernatant was removed and the crystals were washed with 0.5 mL cold *n*-hexane to yield **1-Ga** (123 mg, 0.276 mmol, 53%). ^1^H NMR (500 MHz, toluene-*d*_8_, 26 °C): δ 1.77 (s, 30H, C_5_ Me_5_), −0.30 (s, 12H, GaMe_4_) ppm. ^13^C­{^1^H} NMR (126 MHz, toluene-*d*_8_, 26 °C): δ 120.3 (C_5_Me_5_), 12.0 (C_5_ Me_5_) ppm. The ^13^C­{^1^H} NMR signal of the methyl groups bound to scandium and gallium could not be resolved. ^45^Sc NMR (122 MHz, toluene-*d*_8_, 26 °C): δ 117.3, 92.5 ppm. C_24_H_42_Al_0.3_Ga_0.7_Sc (432.45 g/mol): calcd. (%) C 66.66, H 9.79; found C 66.12, H 9.46. ICP-OES: Ga:Al = 2.75:1.

### Cp*_2_Sc­(InMe_4_) (1-In)

A solution of InMe_3_ (150 mg, 0.938 mmol) and Et_2_O (41 mg, 0.55 mmol) in 2 mL toluene was added at −40 °C to a cooled, stirred solution of **1-Al** (126 mg, 0.313 mmol) in 3 mL toluene. The mixture became slightly cloudy but went back to clear after 5 min during warming to ambient temperature. The resulting yellow solution was concentrated to 2 mL and cooled to −40 °C for crystallization. After 2 days at −40 °C, colorless crystals formed. The supernatant was removed and the crystals were washed with 0.5 mL cold *n*-hexane to yield **1-In** with cocrystallized InMe_3_ as evidenced by SC-XRD and NMR spectra (127 mg, 0.241 mmol, 77%). ^1^H NMR (300 MHz, C_6_D_6_, 26 °C): δ 1.78 (s, 30H, C_5_ Me_5_), −0.24 (s, br, 21H, InMe_4_, InMe_3_) ppm. ^13^C­{^1^H} NMR (75 MHz, C_6_D_6_, 26 °C): δ 120.3 (C_5_Me_5_), 12.0 (C_5_ Me_5_), 2.4 (InMe_4_, InMe_3_) ppm. ^45^Sc NMR (122 MHz, toluene-*d*_8_, 26 °C): δ 130.8, 92.6 ppm.

Prolonged evacuation of **1-In** led to complete removal of cocrystallized InMe_3_ and colorless crystals (102 mg, 0.228 mmol, 73%). ^1^H NMR (400 MHz, C_6_D_6_, 26 °C): δ 1.79 (s, 30H, C_5_ Me_5_), 0.08 to −0.81 (br, 12H, InMe_4_) ppm. ^45^Sc­{^1^H} NMR (97 MHz, C_6_D_6_, 26 °C): δ 179.6, 93.3 ppm. C_24_H_42_Al_0.4_In_0.6_Sc (455.23 g/mol): calcd. (%) C 63.32, H 9.30; found C 64.26, H 9.18. ICP-OES: In:Al = 1.94:1. Although these results are outside the range viewed as establishing analytical purity (C: +0.94%), they are provided to illustrate the best values obtained to date.

### Cp*_6_Sc_4_Ga_8_(CH_2_)_12_Me_6_ (4-Ga)

A solution of **1-Ga** (65 mg, 0.15 mmol) in 0.5 mL C_6_D_6_ was heated to 60 °C for 11 days. During that time, a yellow precipitate formed. The crystalline precipitate was separated from the supernatant and washed with 1 mL *n*-hexane to yield **4-Ga** (24 mg, 0.013 mmol, 35%). C_78_H_132_AlGa_7_Sc_4_ (1764.75 g/mol): calcd. (%) C 53.09, H 7.54; found C 56.69, H 8.05. Although the values are far outside the range viewed as establishing analytical purity (C: +3.60%, H: +0.51%), they are provided to illustrate the best values obtained to date. ICP-OES: Ga:Al = 4.53:1.

### Cp*_6_Sc_4_In_8_(CH_2_)_12_Me_6_ (4-In)

A solution of **1-In** (31 mg, 0.059 mmol) in 0.5 mL C_6_D_6_ was heated to 80 °C for 2 days. During that time, a yellow precipitate formed. The crystalline precipitate was separated from the supernatant and washed with 1 mL *n*-hexane to yield **4-In** (10.3 mg, 5.4 μmol, 37%). C_78_H_132_Al_3_In_5_Sc_4_ (1904.77 g/mol): calcd. (%) C 49.18, H 6.99; found C 49.47, H 6.94. ICP-OES: In:Al = 1.74:1.

### Ga_8_(CH_2_)_12_

Path A: To a solution of **1-Ga** (25 mg, 0.056 mmol) in 0.3 mL C_6_D_6_ was added GaMe_3_ (200 mg, 1.74 mmol). The resulting solution was heated to 100 °C for 4 days, during which a bright yellow precipitate formed. The precipitate was separated from the supernatant and washed with 1 mL toluene and 1 mL *n*-hexane to yield Ga_8_(CH_2_)_12_ (20 mg, 0.028 mmol, 13%). A ^1^H NMR spectrum in THF-*d*_8_ matched the previously published data.^10^ ^45^Sc NMR was silent.

Path B: To a solution of **1-Al** (40 mg, 0.099 mmol) in 4 mL toluene was added GaMe_3_ (115 mg, 1.00 mmol). The resulting solution was heated to 100 °C for 4 days, during which a bright yellow precipitate formed. The precipitate was separated from the supernatant and washed with 1 mL toluene and 3 mL *n*-hexane to yield Ga_8_(CH_2_)_12_ (41.3 mg, 0.0569 mmol, 46%). A ^1^H NMR spectrum in THF-*d*_8_ matched the previously published data (Figure S24). ^45^Sc NMR was silent. ICP-OES: Ga:Al = 8.16:1.

### Cp*_4_Sc_4_In_8_(CH_2_)_12_Me_8_ (5)

Path A: To a solution of **1-In** (32.8 mg, 0.0669 mmol) in 0.3 mL C_6_D_6_ was added a solution of InMe_3_ (107 mg, 0.669 mmol) in 0.3 mL C_6_D_6_. The resulting solution was heated to 100 °C for 3 days, during which a colorless crystalline precipitate formed. The crystalline precipitate was separated from the supernatant and washed with 1 mL toluene and 1 mL *n*-hexane to yield **5** (17 mg, 8.8 μmol, 53%).

Path B: To a solution of **1-Al** (15.6 mg, 0.0388 mmol) in 0.3 mL C_6_D_6_ was added a solution of InMe_3_ (89.3 mg, 0.558 mmol) in 0.3 mL C_6_D_6_. The resulting solution was heated to 100 °C for 3 days, during which a colorless precipitate formed. The crystalline precipitate was separated from the supernatant and washed with 1 mL toluene and 1 mL *n*-hexane to yield **5** (12 mg, 6.2 μmol, 64%). C_60_H_108_In_8_Sc_4_ (1927.89 g/mol): calcd. (%) C 37.38, H 5.65; found C 37.00, H 5.43. ICP-OES: In:Al = 10.07:1.

### Me_2_InCp*InMe_3_ (6)

To a solution of InMe_3_ (65.8 mg, 0.411 mmol) in 0.3 mL C_6_D_6_ was added a solution of HCp* (56.0 mg, 0.411 mmol) in 0.3 mL C_6_D_6_. The resulting solution was heated to 110 °C for 12 days. The volatiles were removed and the remaining solid was dissolved in 4 mL *n*-hexane. After storing the solution at −40 °C for 1 day, colorless single crystals of **6** were obtained (11.9 mg, 27.0 μmol, 7%). ^1^H NMR (400 MHz, C_6_D_6_, 26 °C): δ 1.99 (s, 15H, C_5_ Me_5_), −0.35 (s, br, 15H, InMe) ppm. ^13^C­{^1^H} NMR (101 MHz, C_6_D_6_, 26 °C): δ 115.6 (C_5_Me_5_), 11.3 (C_5_ Me_5_) ppm. The ^13^C­{^1^H} NMR signal of the methyl groups bound to indium could not be resolved. The colorless crystals turned yellow upon prolonged evacuation, and ^1^H NMR spectra indicated loss of InMe_3_. Therefore, elemental analysis was not attempted.

## Supplementary Material



## Notes

Supporting figures, detailed crystallographic data, spectroscopic data (NMR), and analytical details (PDF)
