# Supplementary material for: Scandium—Group 13 Heterobimetallic Methylidene Clusters
Source: Inorg Chem. 2025 Jul 2;64(28):14031–40. doi: 10.1021/acs.inorgchem.5c01520 (PMC12284852; doi:10.1021/acs.inorgchem.5c01520)
Supplement: Supplementary file 1 [file ic5c01520_si_001.pdf]

Supporting Information

Scandium–Group 13 Heterobimetallic Methylidene Clusters

Gernot T. L. Zug, Cäcilia Maichle-Mössmer, and Reiner Anwander*

Institut für Anorganische Chemie, Eberhard Karls Universität Tübingen, Auf der Morgenstelle 18, 72076 Tübingen, Germany

*E-mail for R. A.: reiner.anwander@uni-tuebingen.de

Table of Contents

NMR spectra S3

Crystallographic data and crystal structures S18

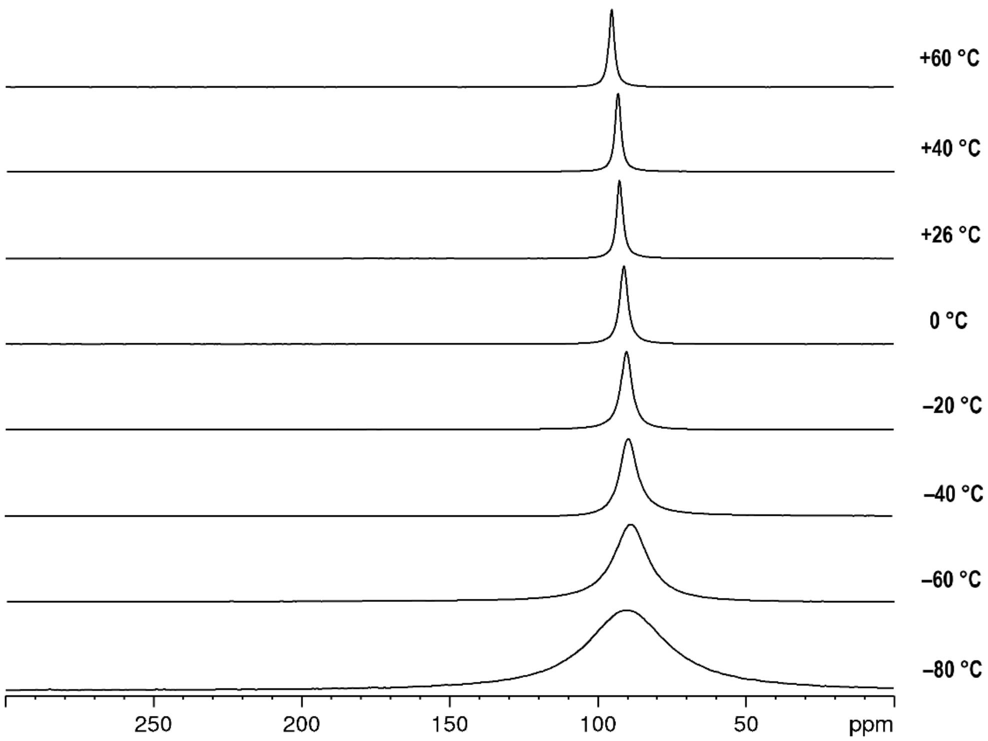

Figure S1. Variable temperature ^{45}Sc NMR spectra (122 MHz) of compound $Cp^*_2ScAlMe_4$ (**1-Al**) in toluene-d_8.

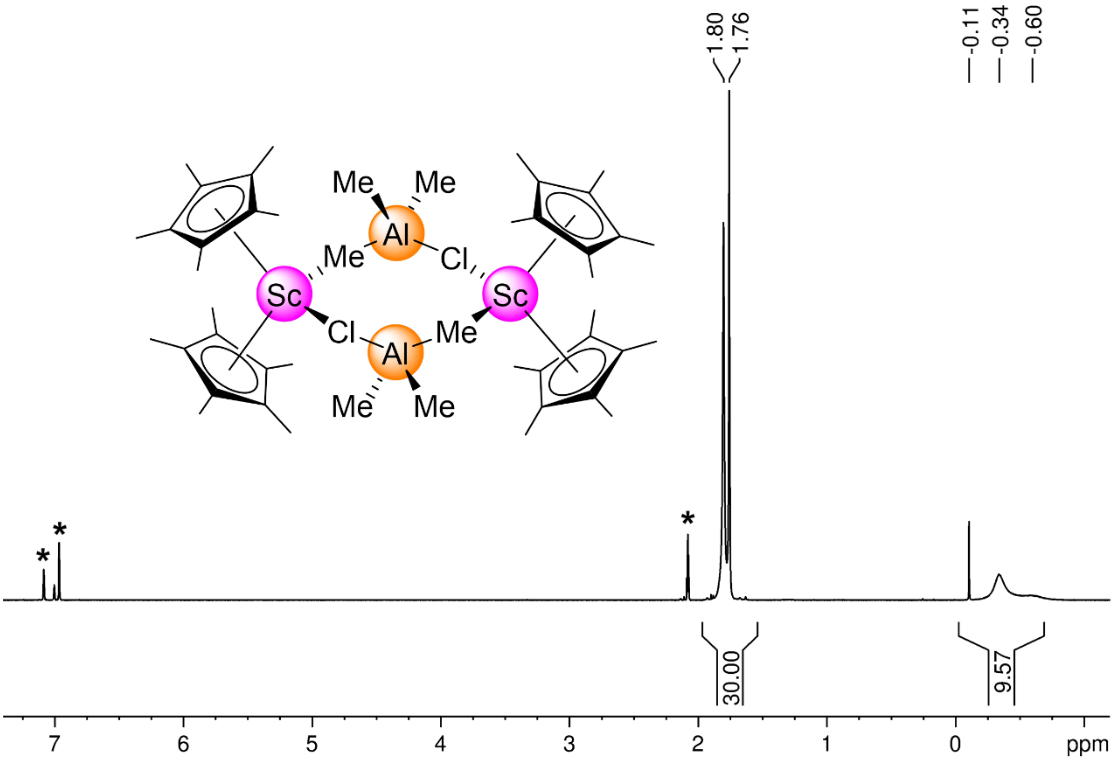

Figure S2. ^{1}H NMR spectrum (500 MHz) of compound $[Cp^*_2ScClAlMe_3]_2$ (**2**) in toluene-d_8 (marked with *) at 26 °C.

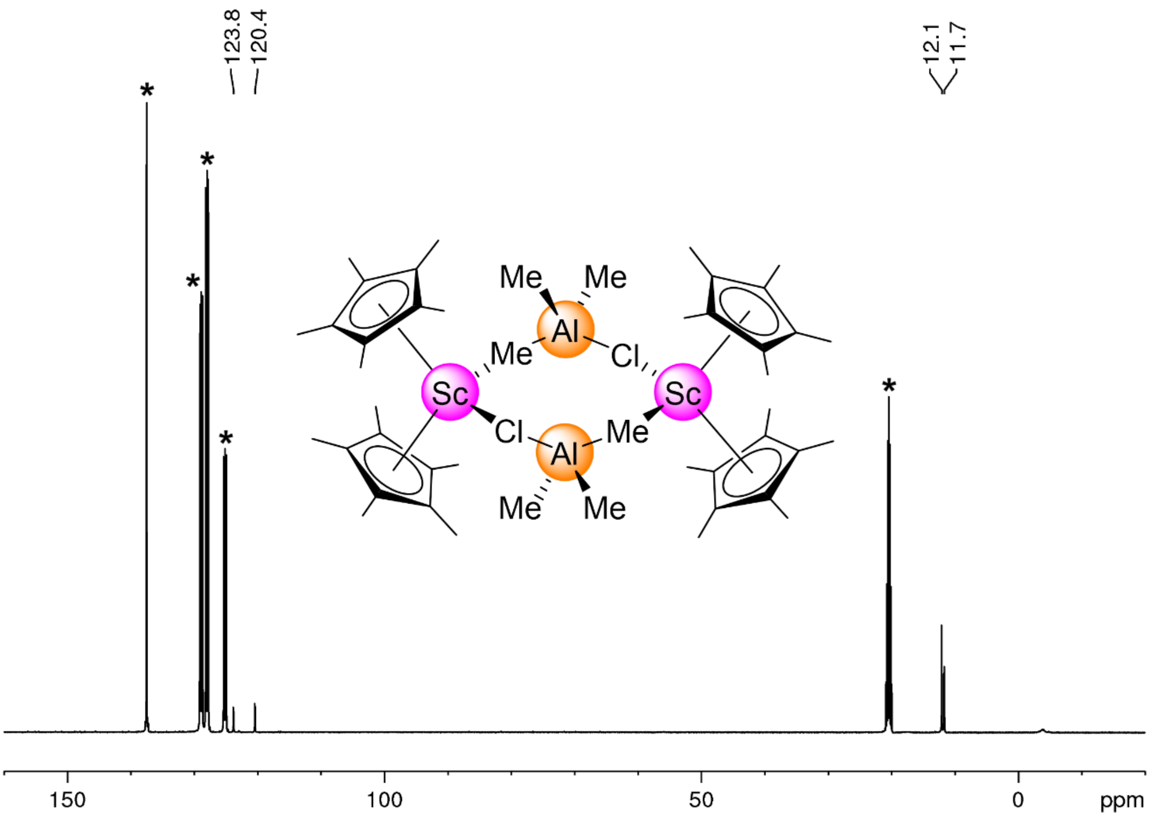

Figure S3. $^{13}C\{^{1}H\}$ NMR spectrum (126 MHz) of compound $[Cp^*_2ScClAlMe_3]_2$ (**2**) in toluene-d_8 (marked with *) at 26 °C.

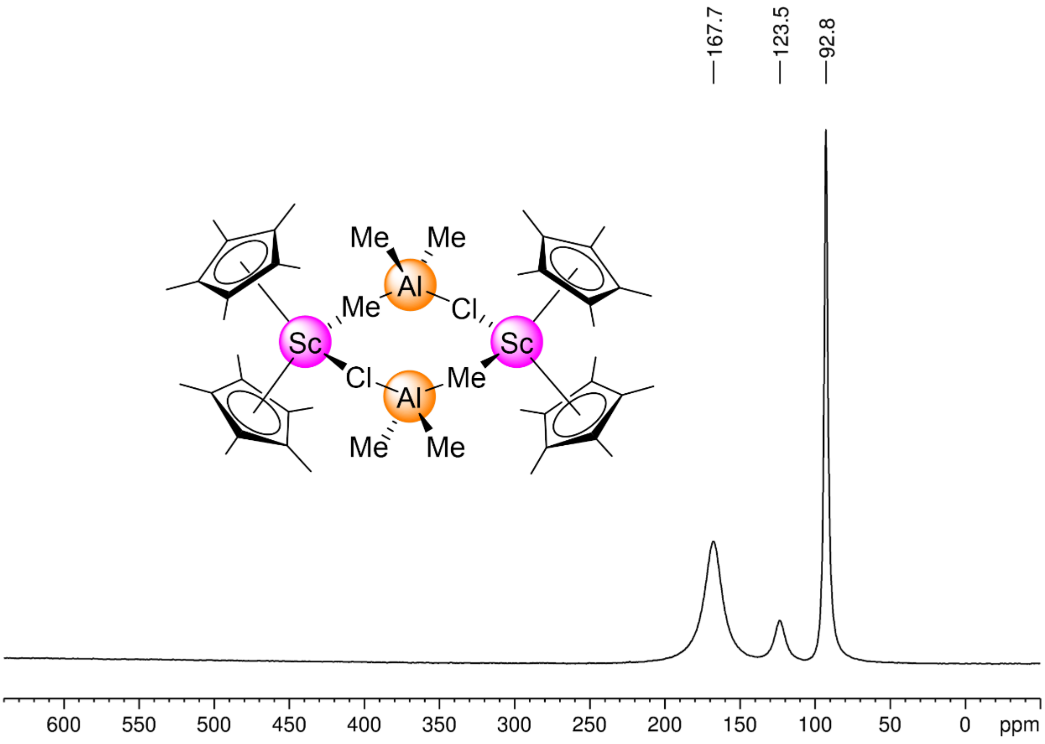

Figure S4. ^{45}Sc NMR spectrum (122 MHz) of compound $[Cp^*_2ScClAlMe_3]_2$ (**2**) in toluene-d_8 at 26 °C.

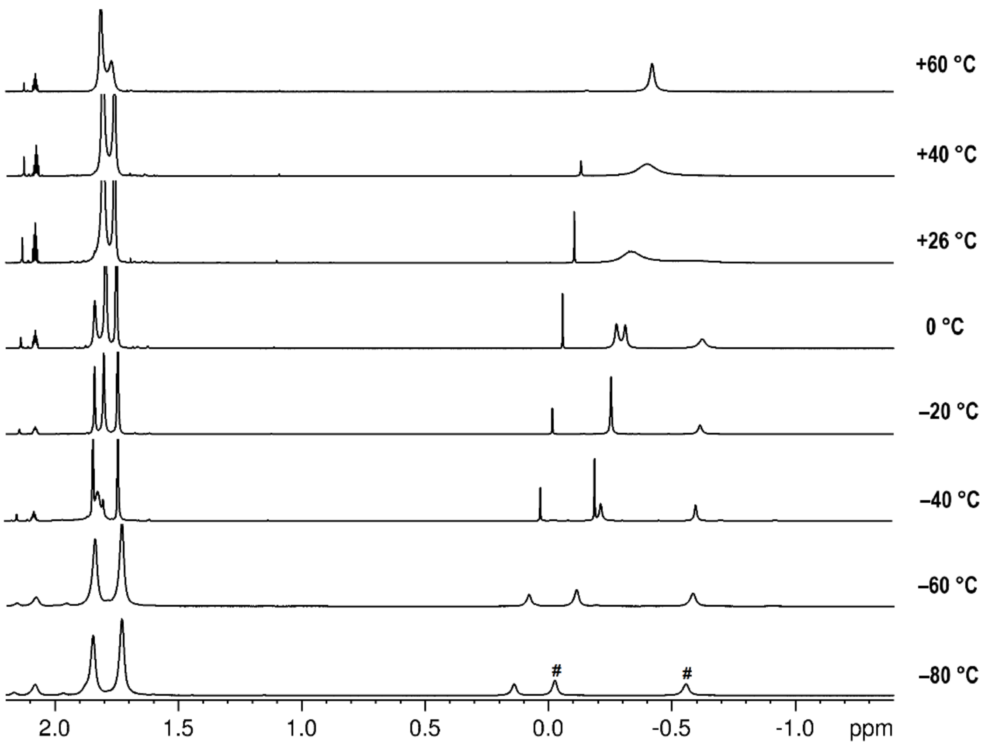

Figure S5. Detail of variable temperature ^{1}H NMR spectra (500 MHz) of compound $[Cp^{*}{}_{2}ScClAlMe_{3}]_{2}$ (**2**) in toluene-d_8. Signals of $Cp^{*}{}_{2}Sc(AlMe_{4})$ are marked with #.

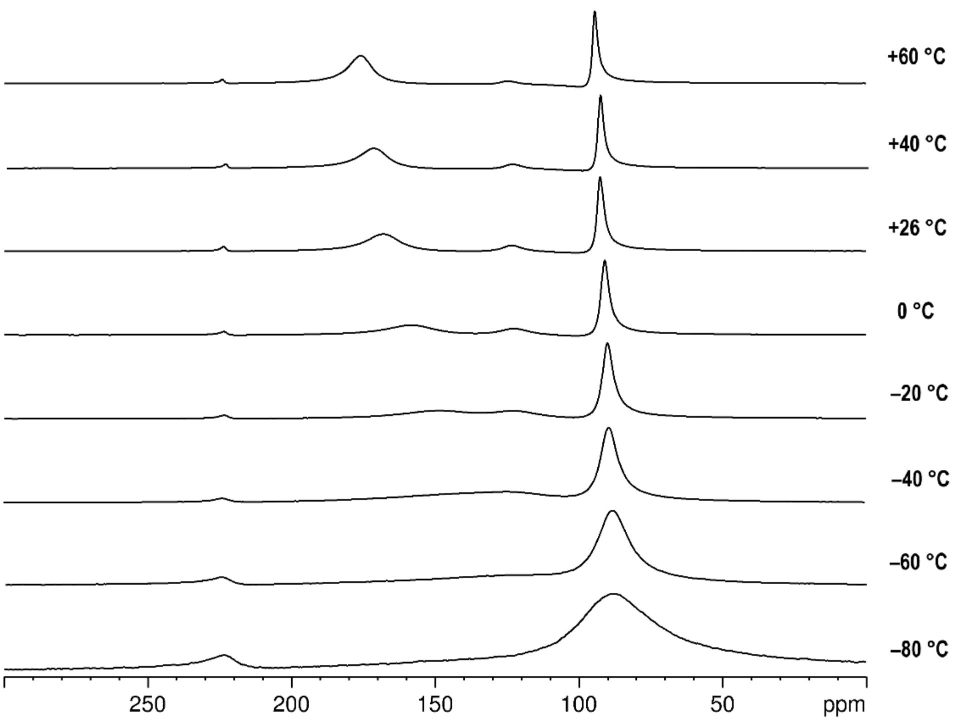

Figure S6. Variable temperature ^{45}Sc NMR spectra (122 MHz) of compound $[Cp^{*}{}_{2}ScClAlMe_{3}]_{2}$ (**2**) in toluene-d_8.

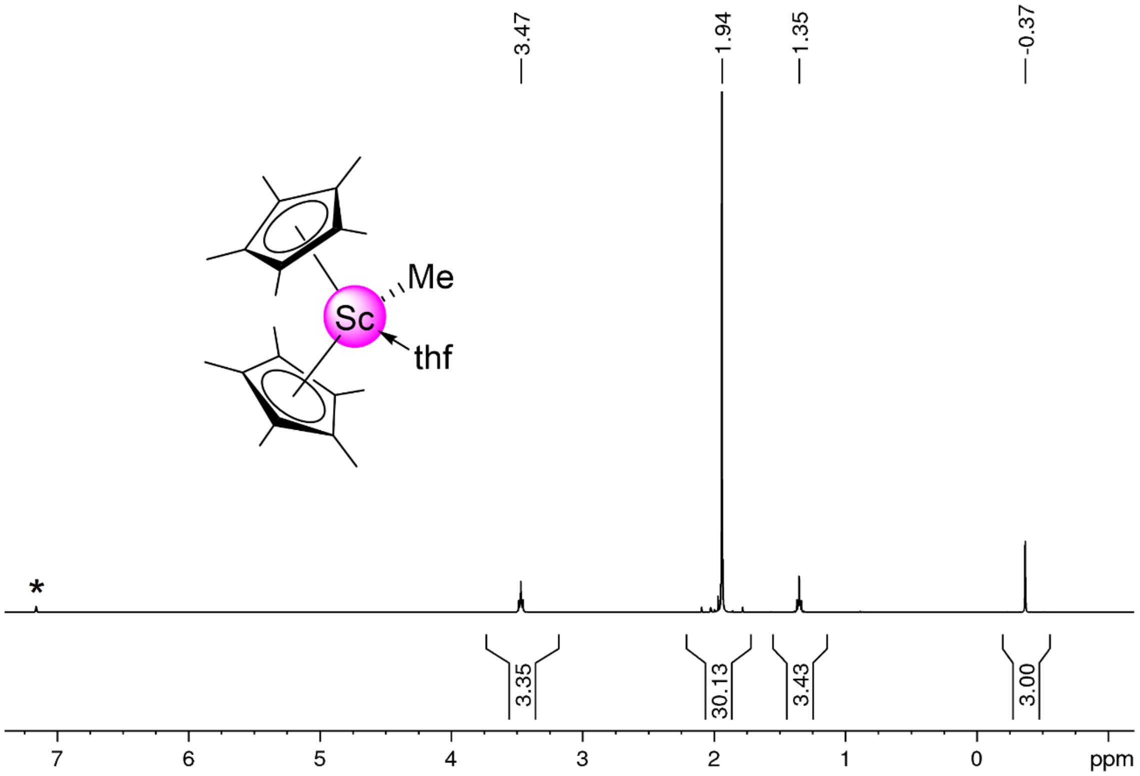

Figure S7. ^{1}H NMR spectrum (400 MHz) of compound Cp*$_2$ScMe(thf) (**3^{thf}**) in C_6D_6 (marked with *) at 26 °C.

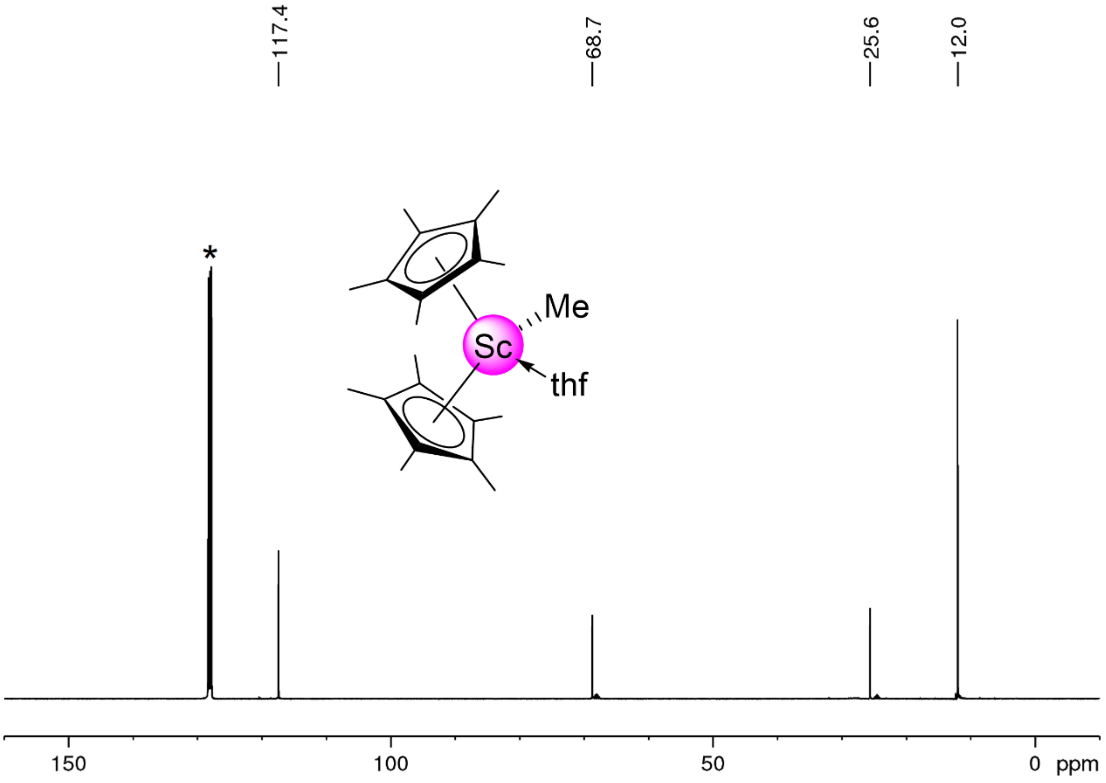

Figure S8. ^{13}C{^{1}H} NMR spectrum (101 MHz) of compound Cp*$_2$ScMe(thf) (**3^{thf}**) in C_6D_6 (marked with *) at 26 °C.

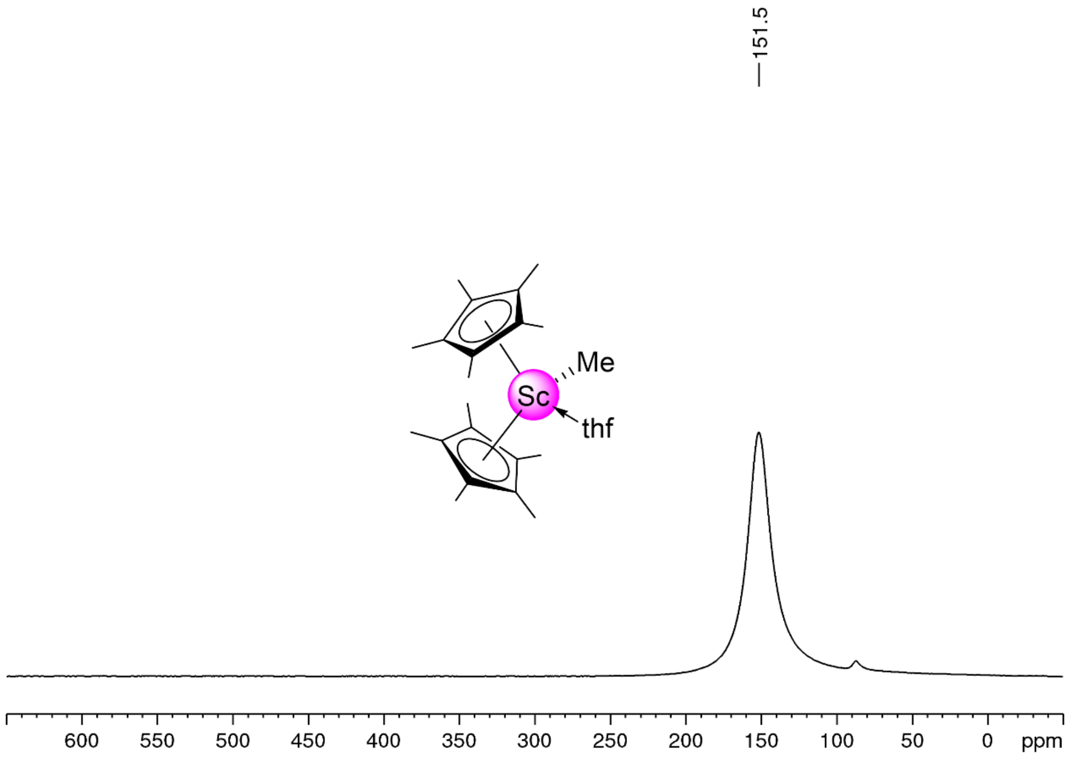

Figure S9. ^{45}Sc{^{1}H} NMR spectrum (97 MHz) of compound Cp*$_2$ScMe(thf) (**3^{thf}**) in C_6D_6 at 26 °C.

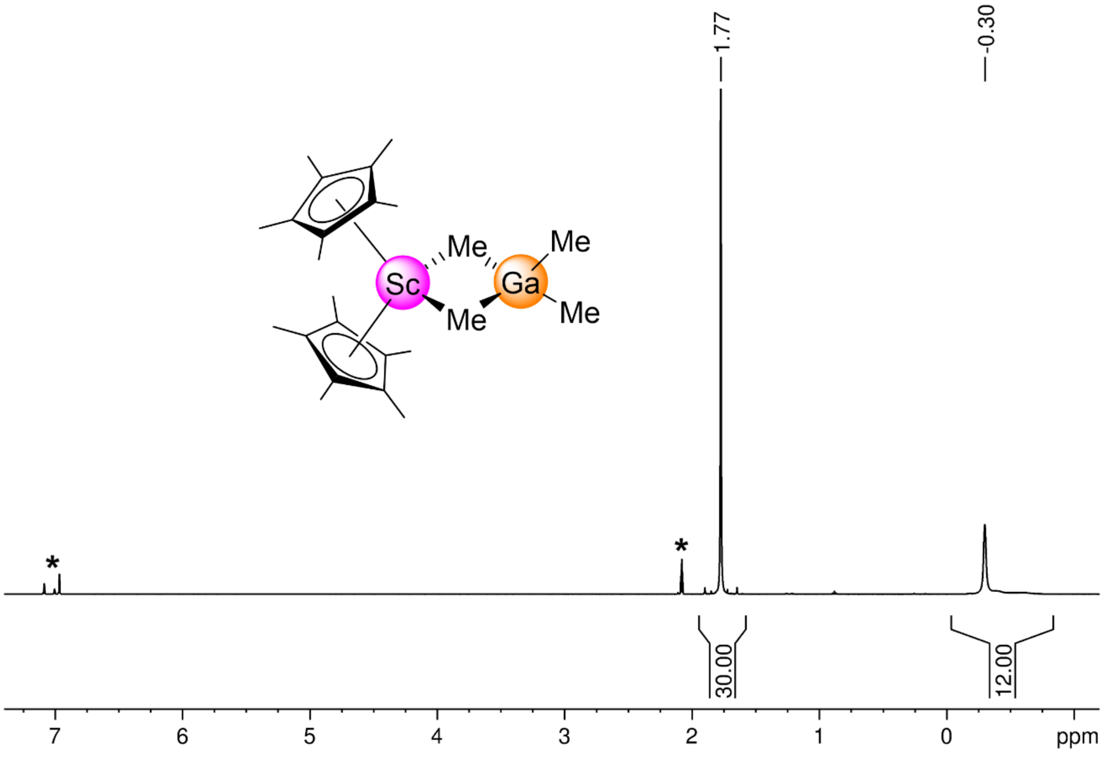

Figure S10. ^{1}H NMR spectrum (500 MHz) of compound Cp*$_2$ScGaMe$_4$ (**1-Ga**) in toluene-d_8 (marked with *) at 26 °C.

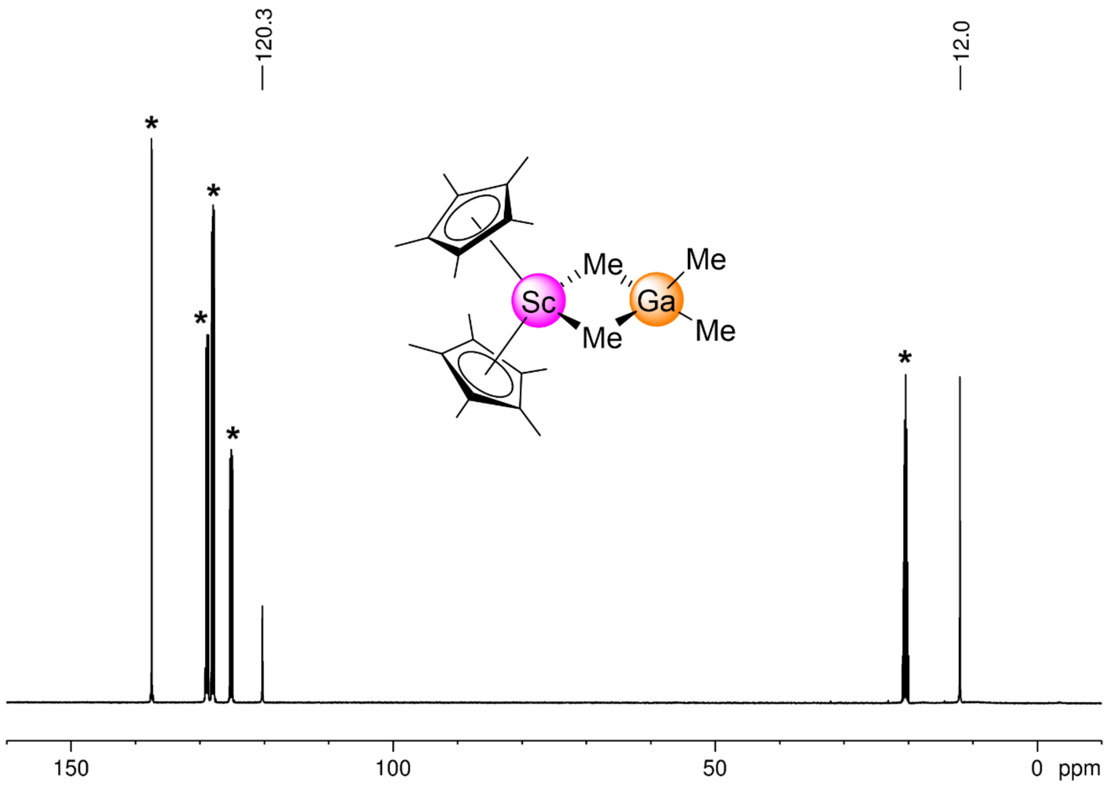

Figure S11. $^{13}C\{^1H\}$ NMR spectrum (126 MHz) of compound Cp*$_2$ScGaMe$_4$ (**1-Ga**) in toluene-d_8 (marked with *) at 26 °C.

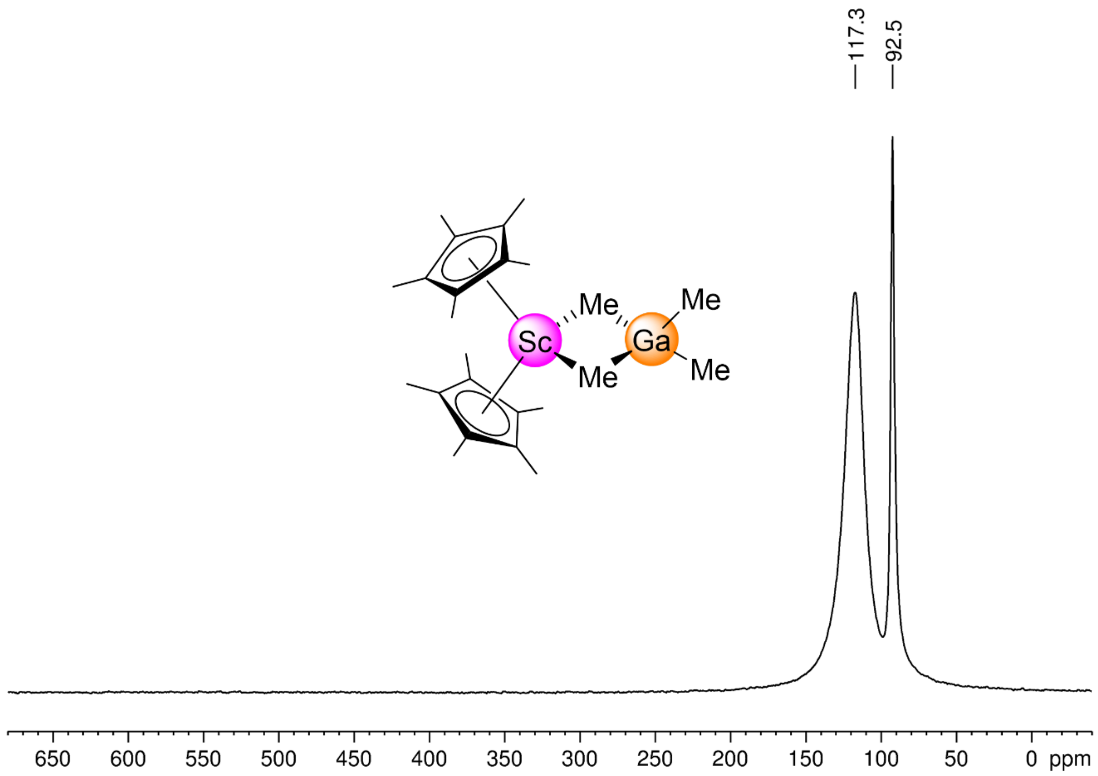

Figure S12. ^{45}Sc NMR spectrum (122 MHz) of compound Cp*$_2$ScGaMe$_4$ (**1-Ga**) in toluene-d_8 at 26 °C.

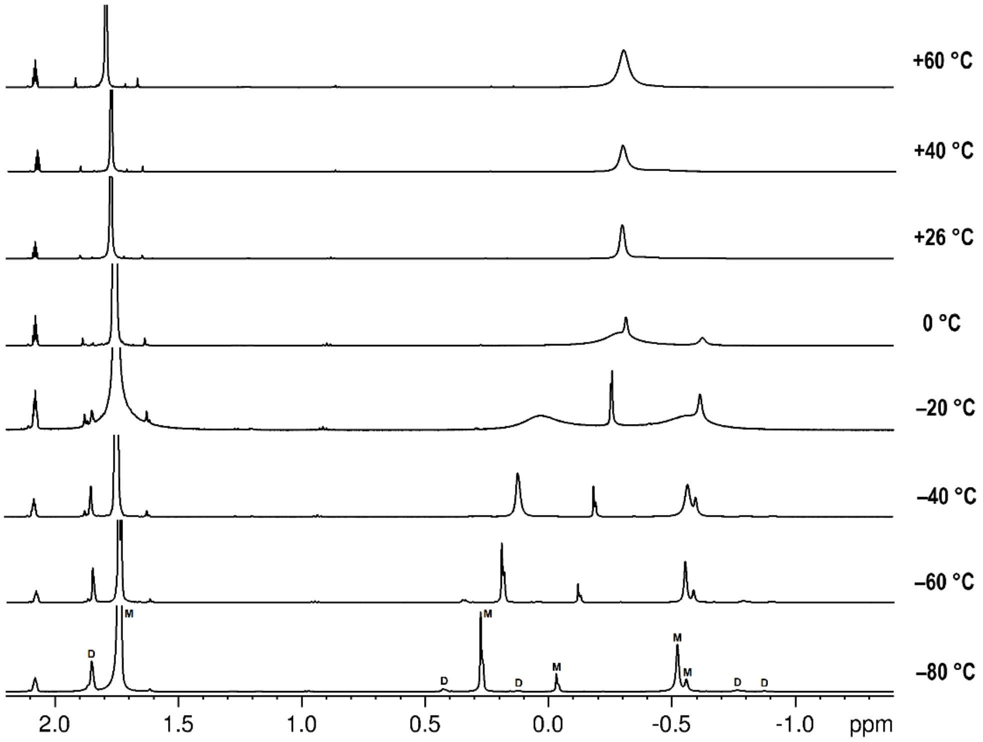

Figure S13. Detail of variable temperature ^{1}H NMR spectra (500 MHz) of compound Cp*$_2$ScGaMe$_4$ (**1-Ga**) in toluene-d_8. Cp*$_2$ScGaMe$_4$ and Cp*$_2$ScAlMe$_4$ show signals of monomers (marked with M) and signals of dimers (marked with D).

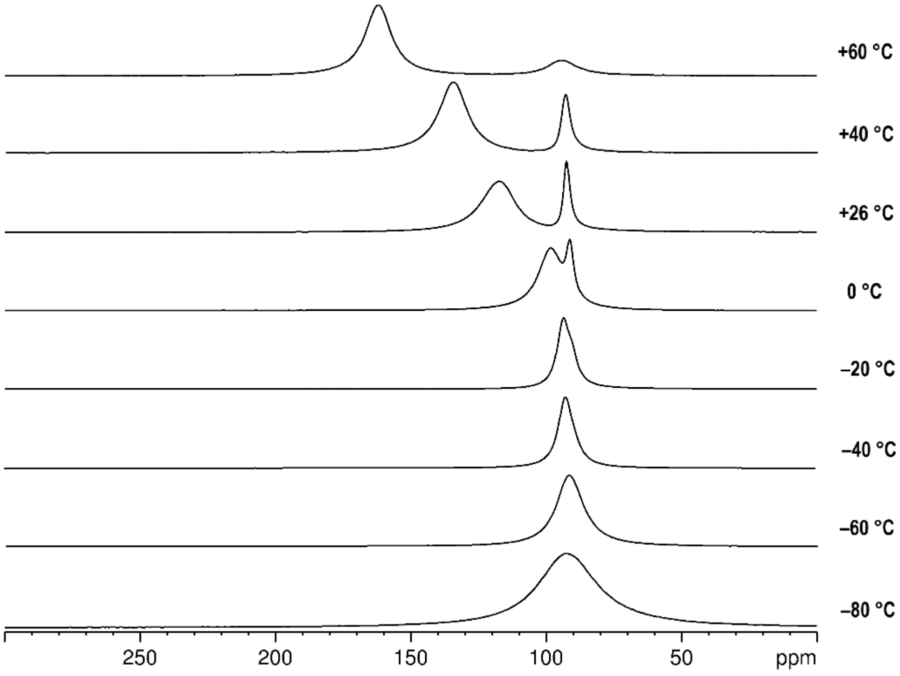

Figure S14. Variable temperature ^{45}Sc NMR spectra (122 MHz) of compound Cp*$_2$ScGaMe$_4$ (**1-Ga**) in toluene-d_8.

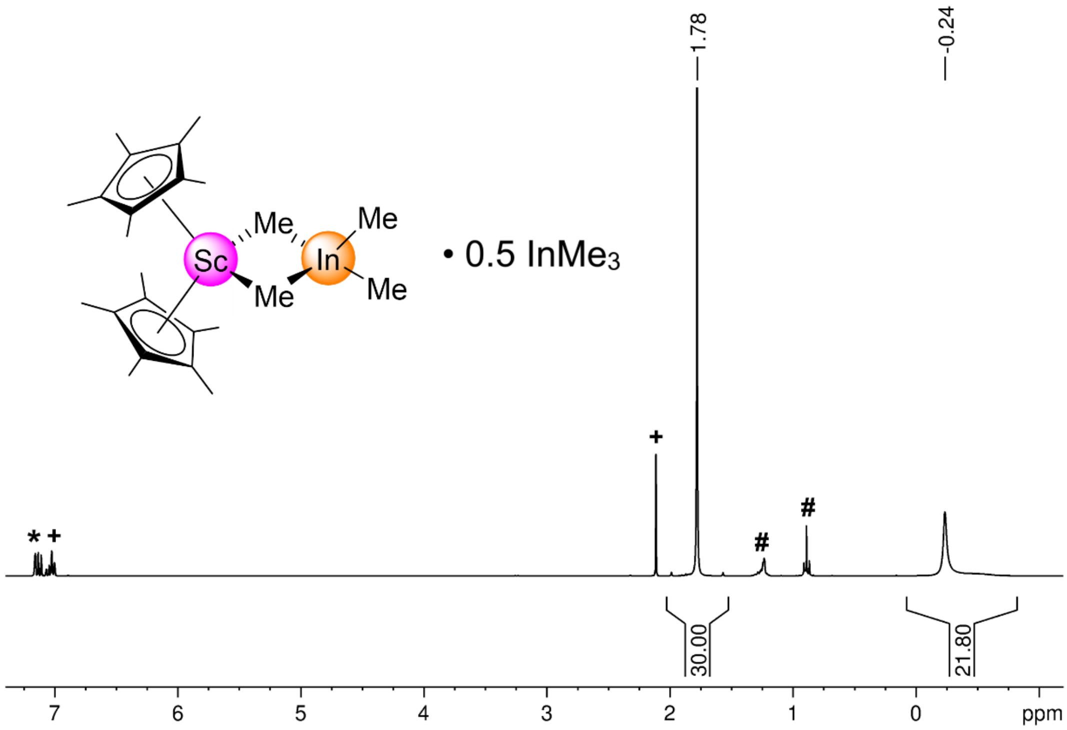

Figure S15. ^{1}H NMR spectrum (300 MHz) of compound Cp*$_2$ScInMe$_4$ (**1-In**) with cocrystallized InMe$_3$ in C$_6$D$_6$ (marked with *) at 26 °C. Residual toluene is marked with +. Residual *n*-hexane is marked with #.

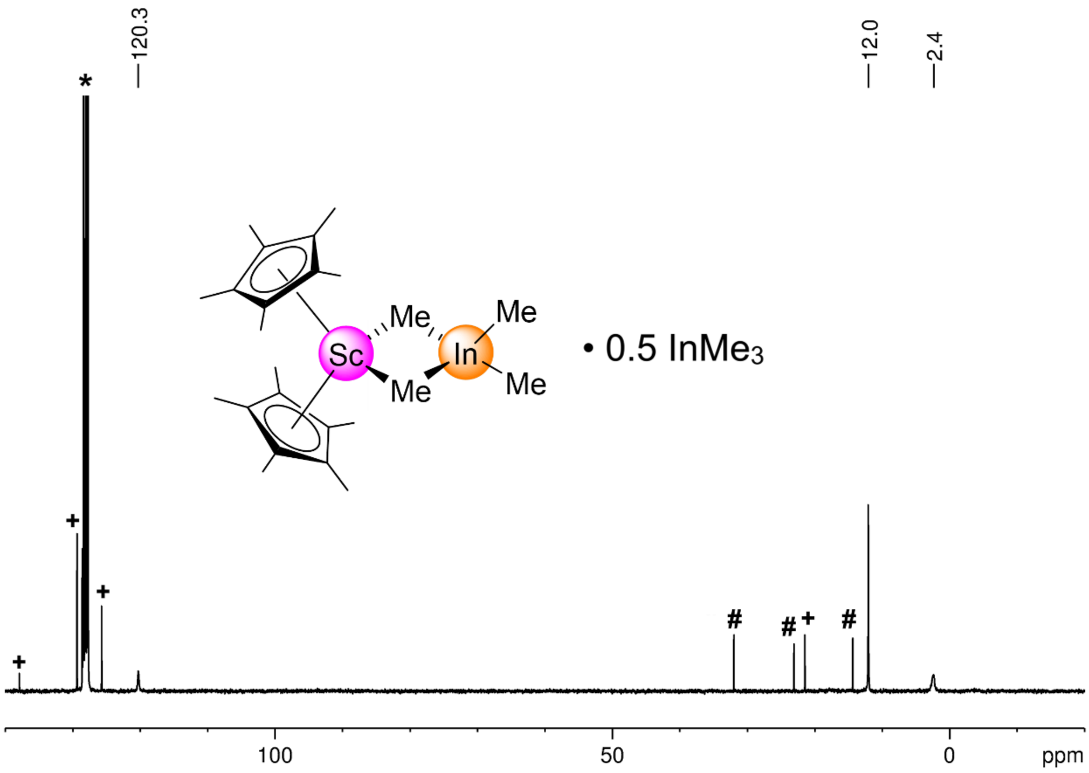

Figure S16. ^{13}C{^{1}H} NMR spectrum (75 MHz) of compound Cp*$_2$ScInMe$_4$ (**1-In**) with co-crystallized InMe$_3$ in C$_6$D$_6$ (marked with *) at 26 °C. Residual toluene is marked with +. Residual *n*-hexane is marked with #.

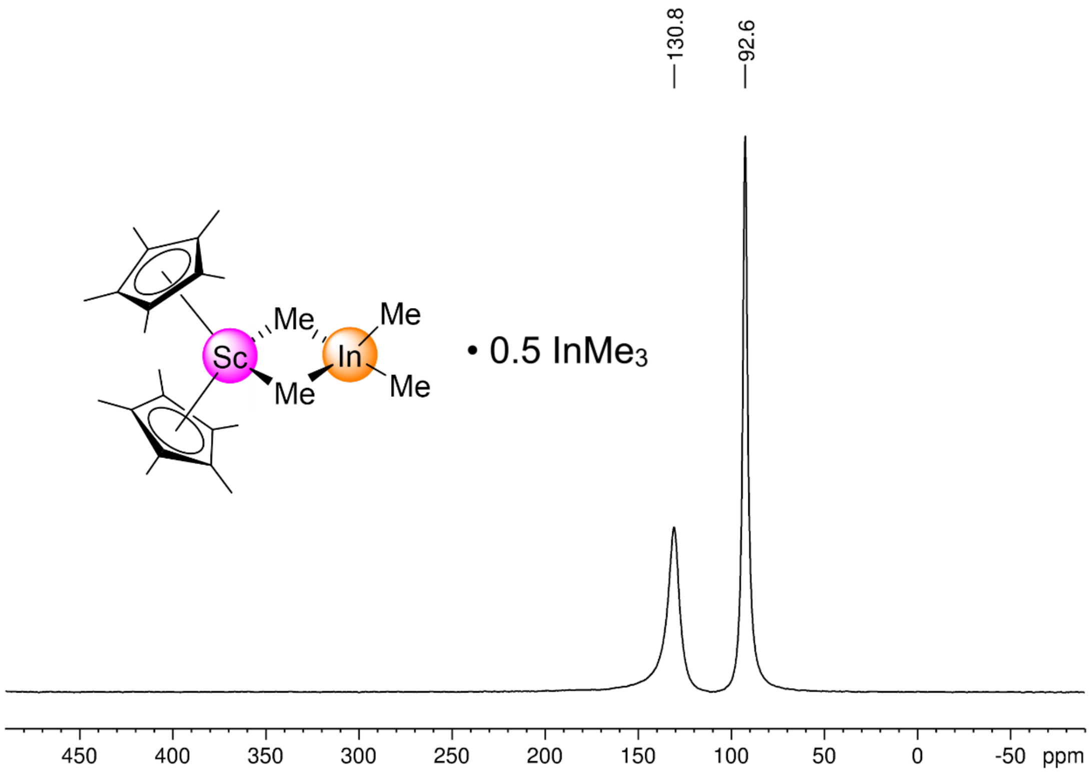

Figure S17. ^{45}Sc NMR spectrum (122 MHz) of compound Cp*$_2$ScInMe$_4$ (**1-In**) with co-crystallized InMe$_3$ in toluene-d_8 at 26 °C.

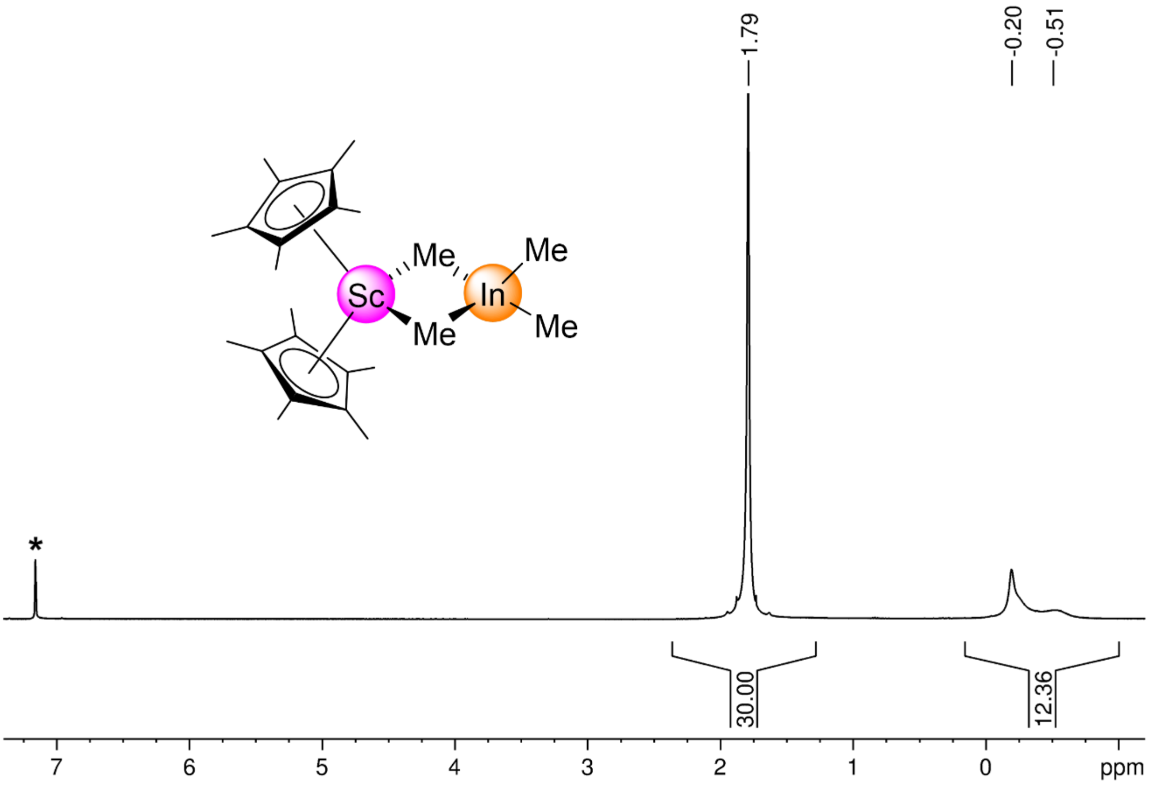

Figure S18. ^{1}H NMR spectrum (400 MHz) of compound Cp*$_2$ScInMe$_4$ (**1-In**) in C_6D_6 (marked with *) at 26 °C.

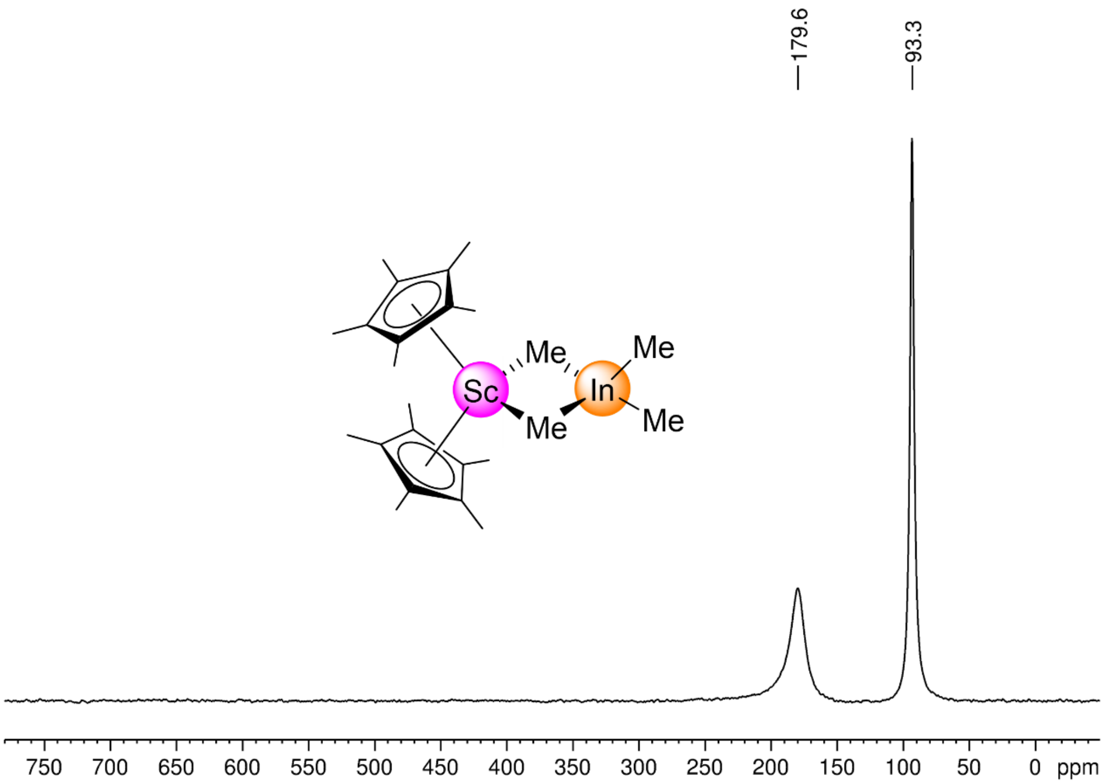

Figure S19. $^{45}Sc\{^{1}H\}$ NMR spectrum (97 MHz) of compound $Cp^*_2ScInMe_4$ (**1-In**) in C_6D_6 at 26 °C.

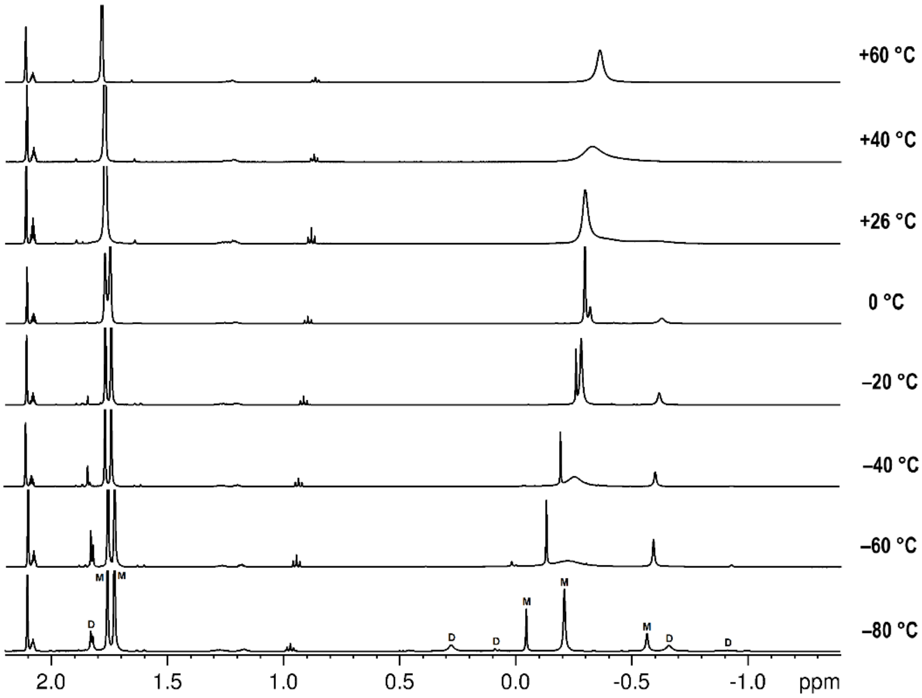

Figure S20. Detail of variable temperature ^{1}H NMR spectra (500 MHz) of compound $Cp^*_2ScInMe_4$ (**1-In**) with co-crystallized $InMe_3$ in toluene-d_8. $Cp^*_2ScInMe_4$ and $Cp^*_2ScAlMe_4$ show signals of monomers (marked with M) and signals of dimers (marked with D).

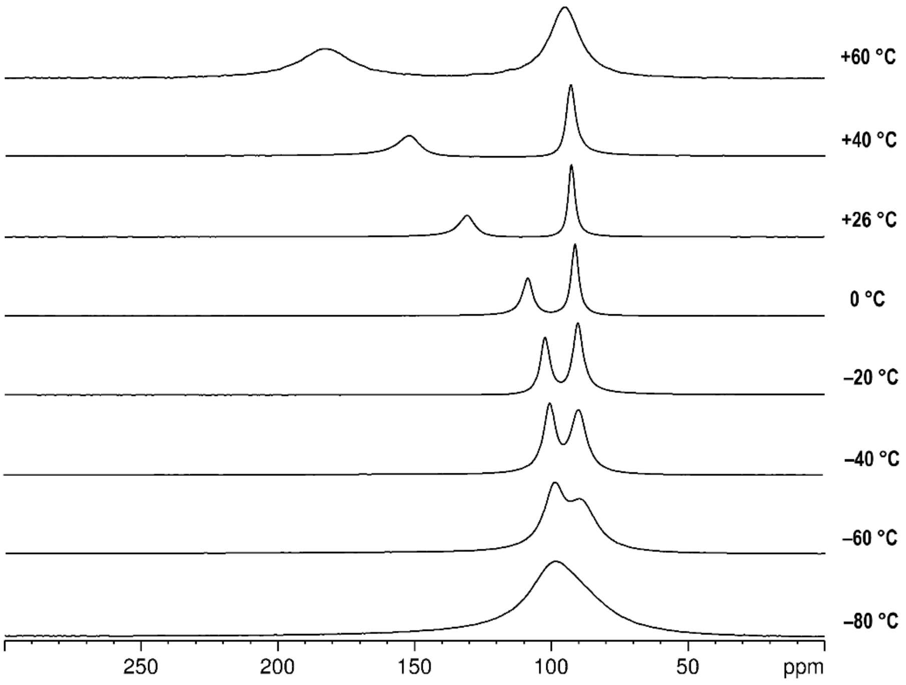

Figure S21. Variable temperature ^{45}Sc NMR spectra (122 MHz) of compound $Cp^*_2ScInMe_4$ (**1-In**) with co-crystallized $InMe_3$ in toluene-d_8.

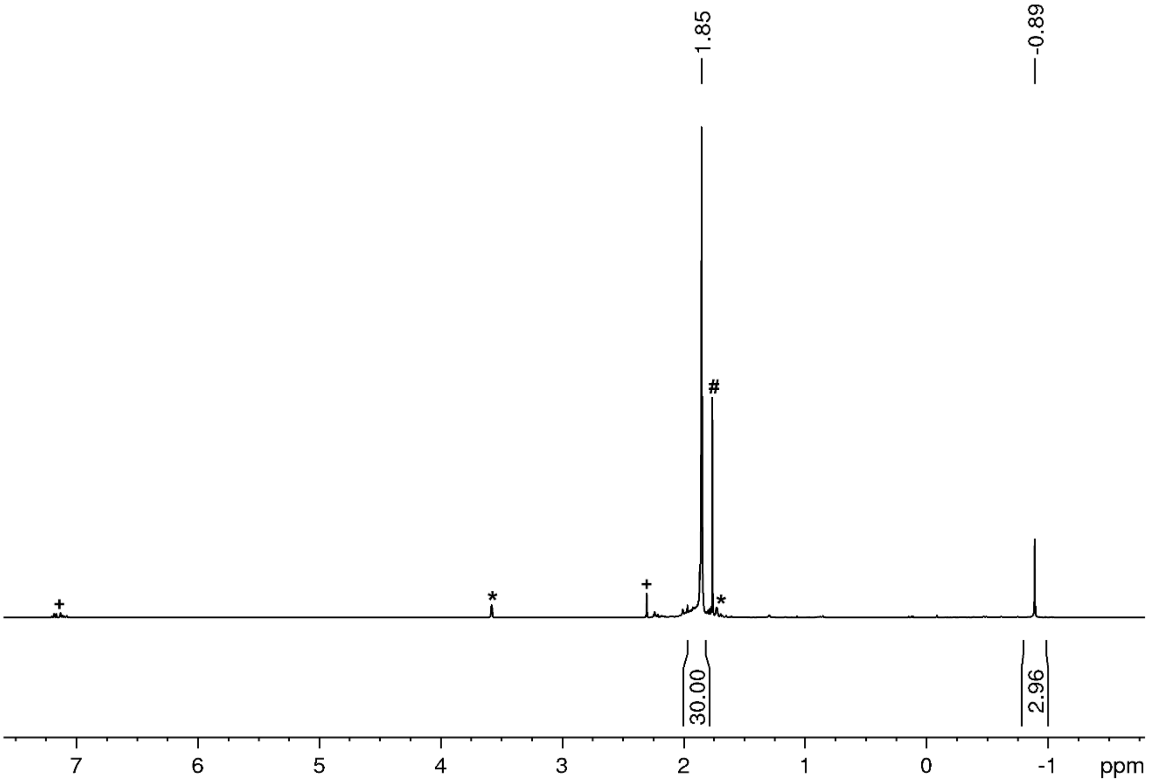

Figure S22. ^1H NMR spectrum (400 MHz) of compound **4-Ga** in THF-d_8 (marked with *) forming $Cp^*_2ScMe(thf)$ at 26 °C. Residual toluene is marked with +. Unknown byproducts are marked with #.

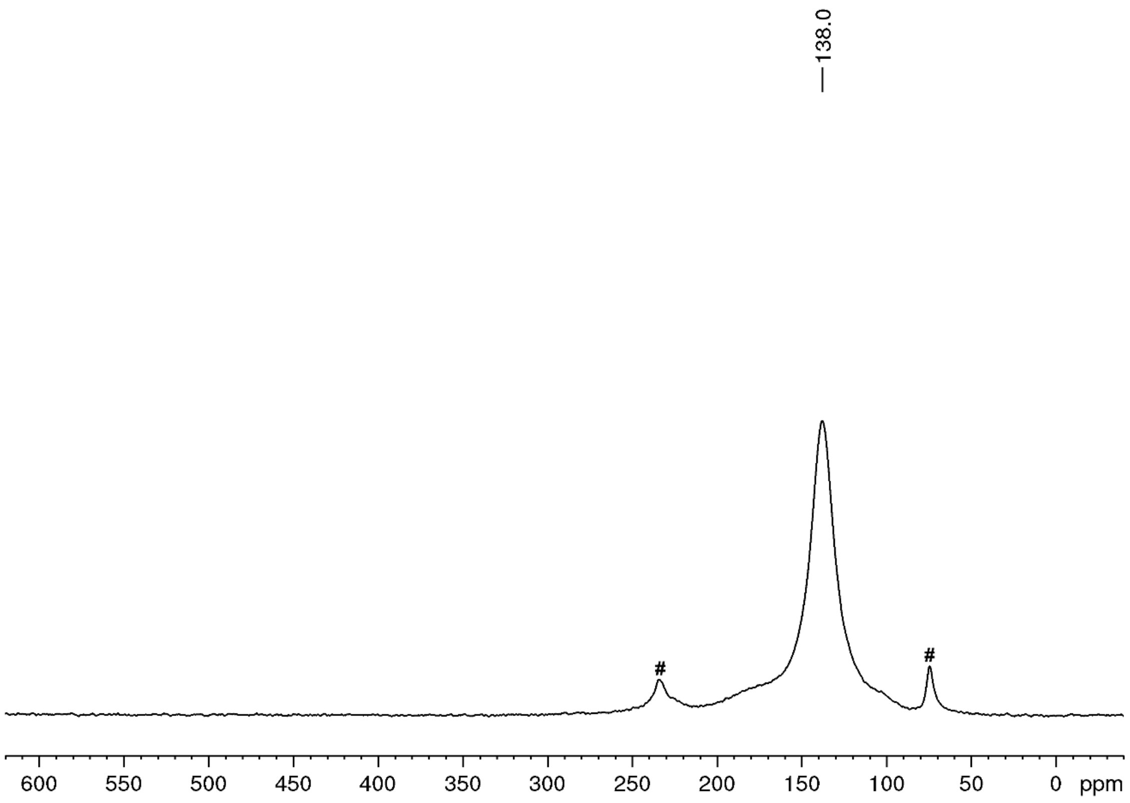

Figure S23. $^{45}Sc\{^1H\}$ NMR spectrum (97 MHz) of compound **4-Ga** in THF-*d*$_8$ forming $Cp^*{}_2ScMe(thf)$ at 26 °C. Unknown byproducts are marked with #.

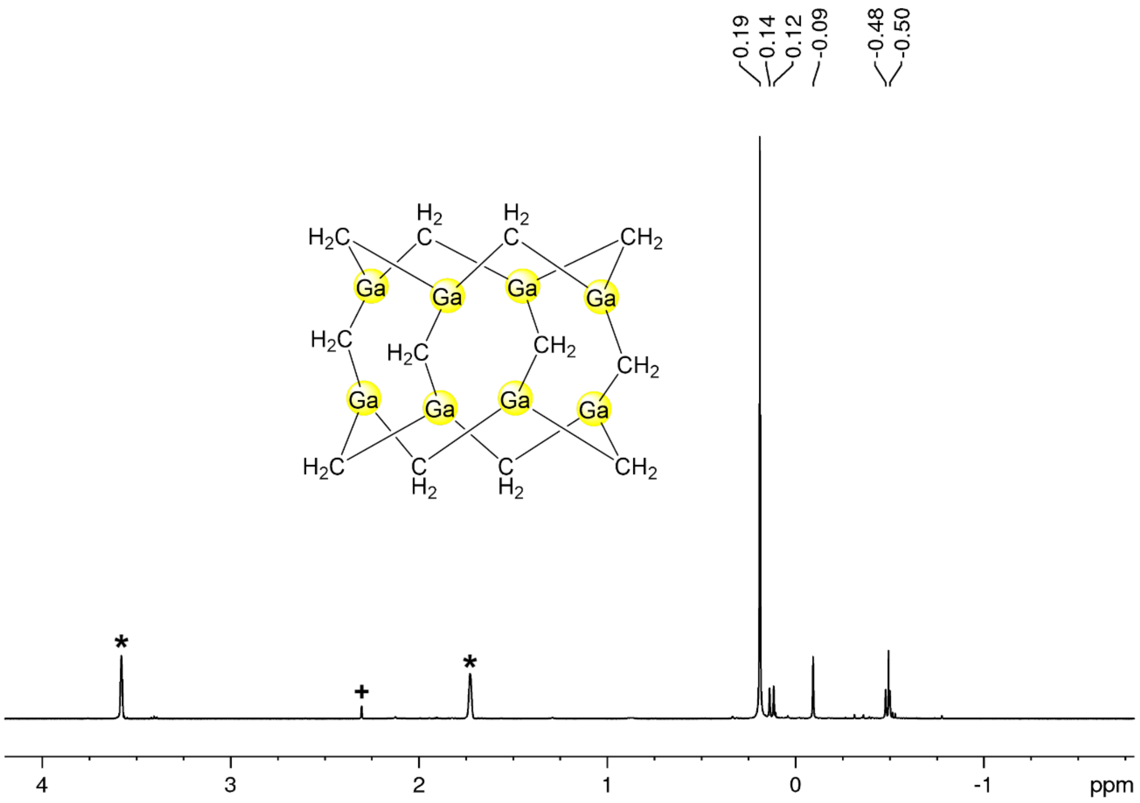

Figure S24. ^1H NMR spectrum (400 MHz) of compound $Ga_8(CH_2)_{12}$ in THF-*d*$_8$ (marked with *) at 26 °C. Residual toluene is marked with +.

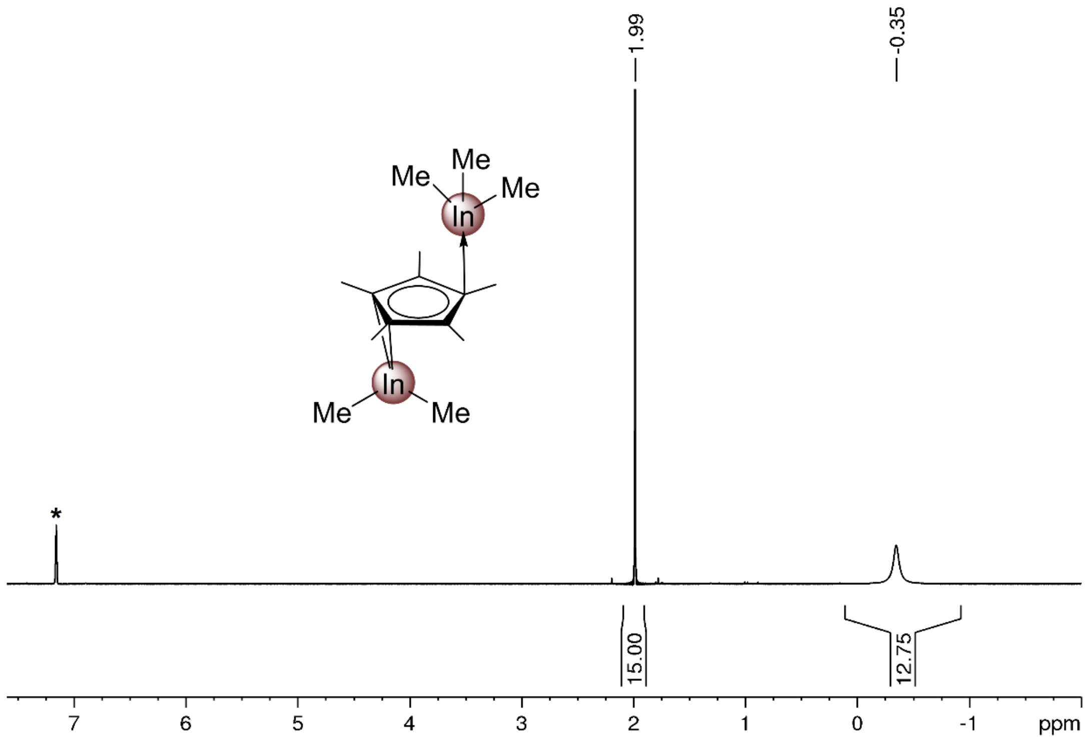

Figure S25. ^{1}H NMR spectrum (400 MHz) of compound $Me_2InCp^*InMe_3$ (**6**) in C_6D_6 (marked with *) at 26 °C.

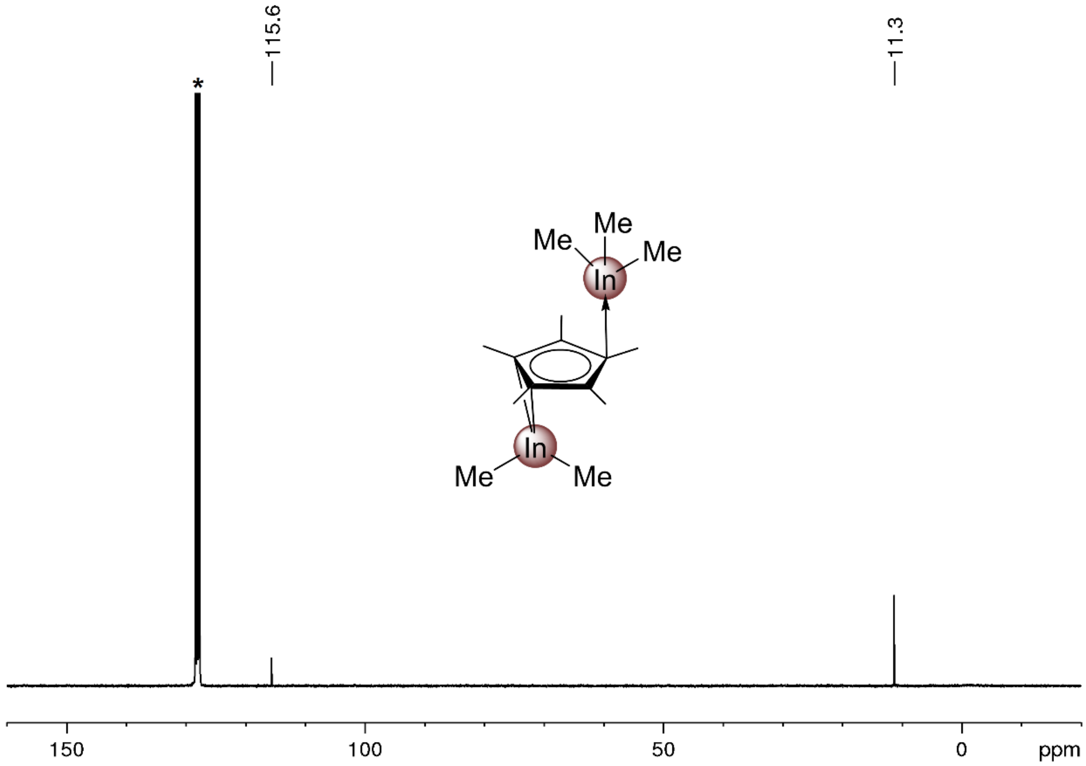

Figure S26. $^{13}C\{^1H\}$ NMR spectrum (101 MHz) of compound $Me_2InCp^*InMe_3$ (**6**) in C_6D_6 (marked with *) at 26 °C.

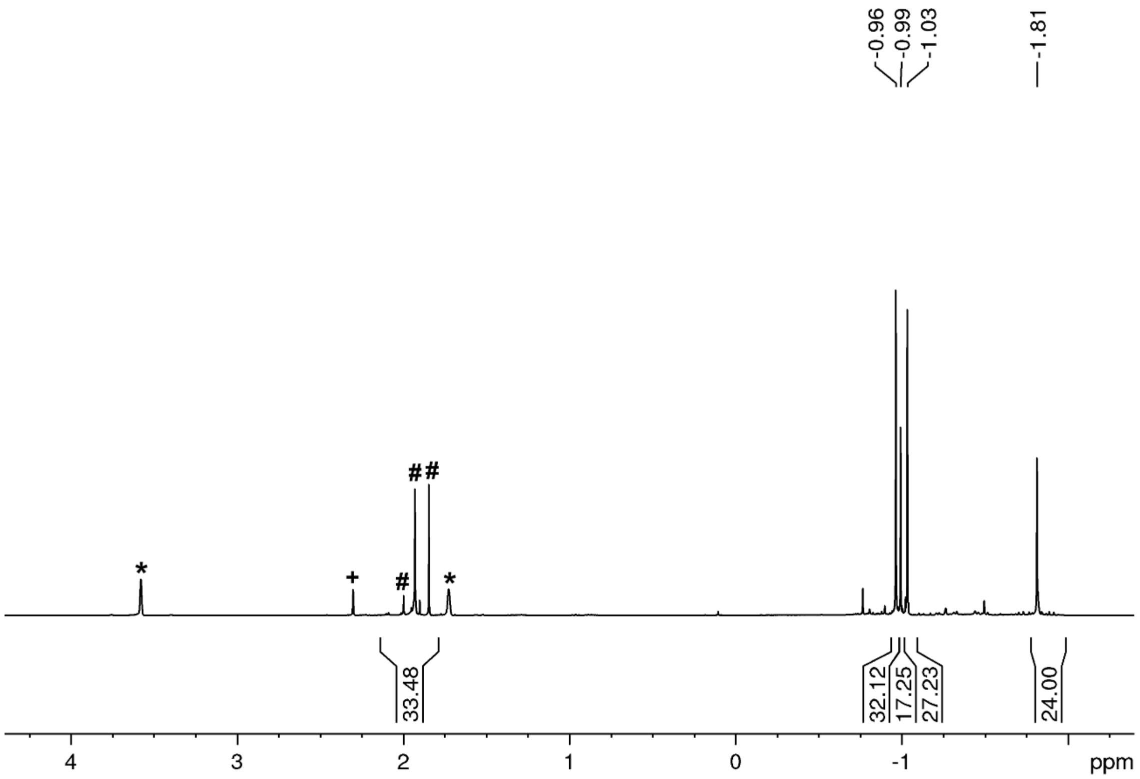

Figure S27. ^{1}H NMR spectrum (400 MHz) of the product of **1-Al** with 100 equiv. $AlMe_3$ in THF-d_8 (marked with *) at 26 °C. Residual toluene is marked with +. Cp* signals are marked with #.

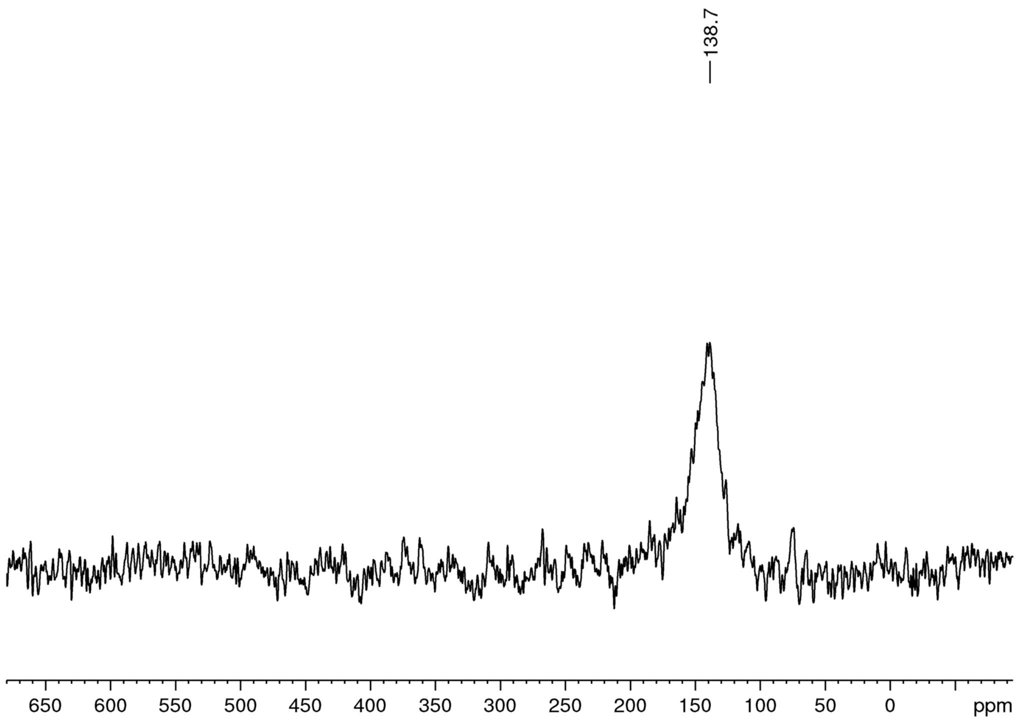

Figure S28. ^{45}Sc{^{1}H} NMR spectrum (97 MHz) of the product of **1-Al** with 100 equiv. $AlMe_3$ in THF-d_8 at 26 °C.

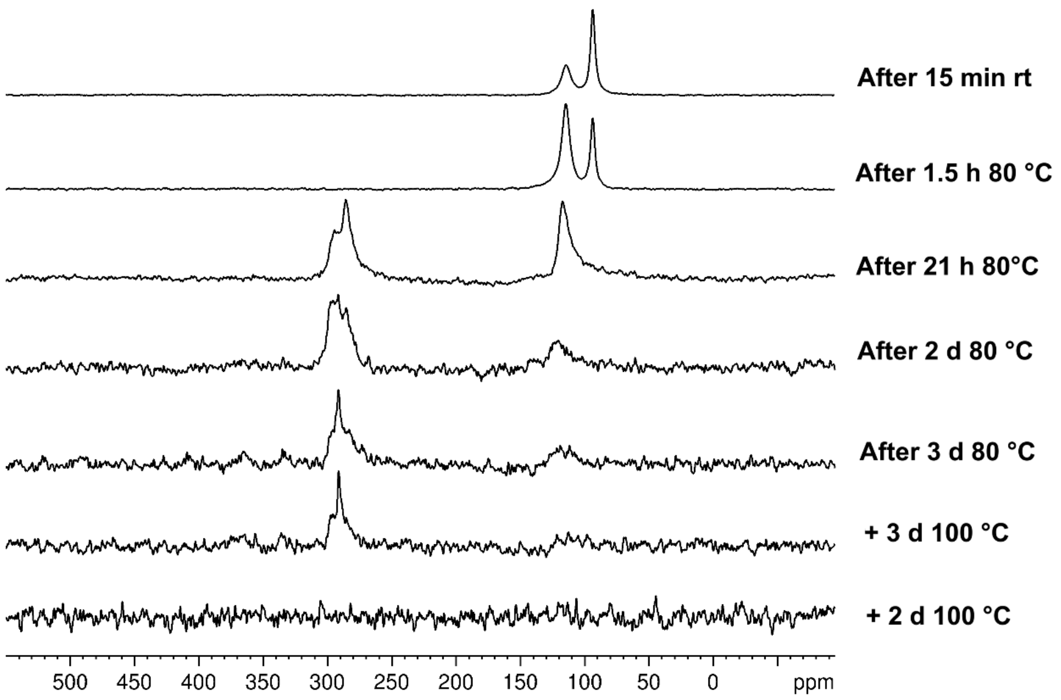

Figure S29. Monitoring of the reaction of $Cp^*_2ScInMe_4$ (**1-In**) with excess $InMe_3$ in a J. Young valved NMR tube without agitation *via* $^{45}Sc\{^1H\}$ NMR spectroscopy (97 MHz) in C_6D_6 at 26 °C to yield $Cp^*_4Sc_4In_8(CH_2)_{12}Me_8$ (**5**).

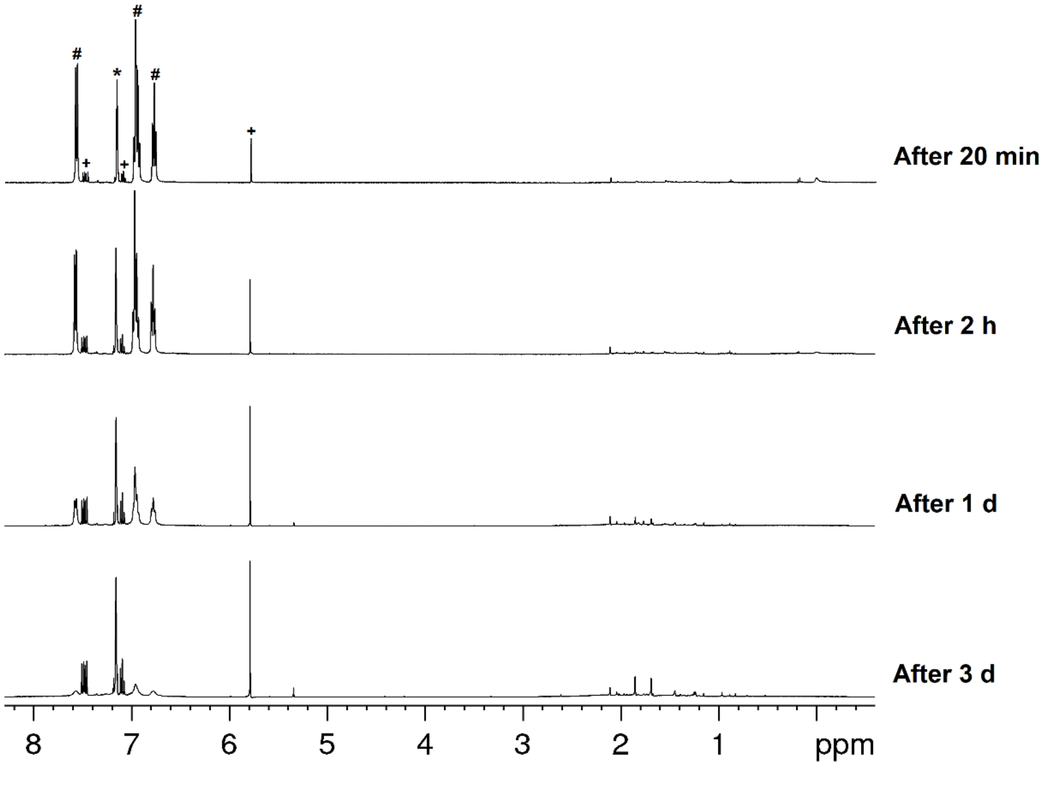

Figure S30. ^1H NMR spectrum (400 MHz) of the reaction mixture of 10.1 mg **5** with 6 equiv. fluorenone (marked with #) over the course of 3 days at ambient temperature in a J. Young-valved NMR tube in C_6D_6 (marked with *) at 26 °C. Dibenzofulvene is marked with +. All other peaks cannot be assigned to a known compound.

Crystal structures and crystallographic data

All crystals except compound **4-E** (E = Ga, In) and **5** were crystallized at −40 °C, using toluene (**2**, **1-Ga**, **1-In**), *n*-pentane (**3**), *n*-hexane (**6**), or mixtures of *n*-hexane/THF (**3^thf^**). Crystals of **4-E** (E = Ga, In) and **5** could be obtained from a hot solution of C_6D_6. For complex **4-Ga** only a connectivity is given. There, it was not possible to isolate a single crystal and to separate reflections for different individuals in the reciprocal space. This may cause the complete disorder of the structure. In **1-In** and **4-In** a substitutional disorder of In and Al could be found in different ranges depending on the position. In **4-In**, for all CH_2 and CH_3 belonging to the In/Al core the hydrogen atoms could be found in the difference Fourier map. For compound **2** a substitutional disorder was also found for Cl/CH_3. These disorders lead to non-integer atom numbers. Further details of the refinement and crystallographic data are listed in Table S1, S2, and S3 and in the CIF files. CCDC depositions 2440710–2440718 contain all the supplementary crystallographic data for this paper. These data can be obtained free of charge from The Cambridge Crystallographic Data Centre via www.ccdc.cam.ac.uk/data_request/cif.

Table S1. Crystallographic data for compounds **2**, **3**, and **3^{thf}**

Compound	**2**	**3**	**3^{thf}**
Formula	$C_{74.8}H_{113}Al_2Cl_{1.2}Sc_2$	$C_{21}H_{33}Sc$	$C_{25}H_{41}OSc$
CCDC	2440713	2440711	2440718
M [g mol^{-1}]	1199.36	330.43	402.54
color/shape	colorless/block	colorless/plate	colorless/block
Crystal dimensions [mm]	0.501 x 0.257 x 0.216	0.212 x 0.166 x 0.095	0.525 x 0.256 x 0.252
cryst. system	Triclinic	Orthorhombic	Monoclinic
space group	$P\bar{1}$	$P2_12_12_1$	$P2_1/c$
a [Å]	11.2036(3)	8.4089(10)	19.5452(16)
b [Å]	12.4184(3)	11.0889(13)	14.9045(8)
c [Å]	14.6076(4)	20.886(3)	17.1958(10)
α [°]	102.6760(10)	90	90
β [°]	100.5200(10)	90	116.032(2)
γ [°]	112.7969(10)	90	90
V [Å^3]	1745.17(8)	1947.6(4)	4501.1(5)
Z	1	4	8
T [K]	100(2)	100(2)	100(2)
wavelength [Å]	0.71073	0.71073	0.71073
ρ_{calcd} [g cm^{-3}]	1.141	1.127	1.188
μ [mm^{-1}]	0.306	0.374	0.339
F(000)	651	720	1760
Θ range [°]	1.497/28.503	2.611/28.743	1.792/30.507
unique reflns	8842	5041	13719
observed reflns	68471	15934	58325
R1[b]/*w*R2(I>2σ)[c]	0.0477/0.1131	0.0440/0.0969	0.0487/0.1270
R1[b]/*w*R2(all data)[c]	0.0597/0.1223	0.0594/0.1055	0.0695/0.1423
GOF[a]	1.031	1.016	1.047

[a]GOF = $[\Sigma w(F_0^2 - F_c^2)^2/(n_0 - n_p)]^{1/2}$. [b]$R_1 = \Sigma(\| F_0\| - \|F_c\|) / \Sigma \|F_0\|$, $F_0 > 4\sigma(F_0)$. [c]$wR_2 = \{\Sigma[w(F_0^2 - F_c^2)^2 / \Sigma[w(F_0^2)^2]\}^{1/2}$.

Table S2. Crystallographic data for compounds **1-Ga**, **1-In**, and **4-Ga**

Compound	**1-Ga**	**1-In**	**4-Ga Connectivity**
Formula	$C_{24}H_{42}GaSc$	$C_{27}H_{51}Al_{0.56}In_{1.44}Sc$ 1.5 C_7H_8	$C_{92}H_{148}Ga_8Sc_4$
CCDC	2440715	2440717	2440712
M [g mol^{-1}]	445.25	739.61	1991.70
color/shape	colorless/block	colorless/block	colorless/block
Crystal dimensions [mm]	0.540 x 0.456 x 0.185	0.447 x 0.420 x 0.246	0.158 x 0.155 x 0.090
cryst. system	Triclinic	Orthorhombic	Triclinic
space group	$P\bar{1}$	*Pbca*	$P\bar{1}$
a [Å]	11.1454(3)	20.6623(7)	11.4714(7)
b [Å]	14.2951(4)	16.1453(5)	14.4371(9)
c [Å]	14.6512(4)	23.0933(8)	15.8359(10)
α [°]	96.6210(10)	90	66.184(2)
β [°]	90.4440(10)	90	70.392(3)
γ [°]	90.2110(10)	90	85.239(3)
V [Å^3]	2318.63(11)	7703.9(4)	2256.0(2)
Z	4	8	1
T [K]	100(2)	100(2)	100(2)
wavelength [Å]	0.71073	0.71073	0.71073
ρ_{calcd} [g cm^{-3}]	1.276	1.275	1.466
μ [mm^{-1}]	1.460	1.068	2.668
F(000)	952	3096	1032
Θ range [°]	1.827/30.528	1.828/30.543	1.490/26.270
unique reflns	14127	11780	9063
observed reflns	111125	117432	52485
R1[b]/*w*R2(I>2σ)[c]	0.0333/0.0957	0.0398/0.0921	0.0770/0.2169
R1[b]/*w*R2(all data)[c]	0.0399/0.0996	0.0495/0.0972	0.1052/0.2443
GOF[a]	1.042	1.128	1.032

[a]GOF = $[\Sigma w(F_0^2 - F_c^2)^2/(n_0 - n_p)]^{1/2}$. [b]$R_1 = \Sigma(| |F_0| - |F_c| |) / \Sigma |F_0|$, $F_0 > 4\sigma(F_0)$. [c]$wR_2 = \{\Sigma[w(F_0^2 - F_c^2)^2/ \Sigma[w(F_0^2)^2]\}^{1/2}$.

Table S3. Crystallographic data for compounds **4-In** and **5**

Compound	**4-In**	**5**	**6**
Formula	$C_{78}H_{132}Al_{3.66}In_{4.34}Sc_4$	$C_{60}H_{108}In_8Sc_4$	$C_{15}H_{30}In_2$
CCDC	2440714	2440710	2456234
M [g mol^{-1}]	1846.73	1927.86	440.03
color/shape	colorless/block	colorless/needle	colorless/needle
Crystal dimensions [mm]	0.091 x 0.089 x 0.081	0.181 x 0.076 x 0.075	0.150 x 0.076 x 0.037
cryst. system	Triclinic	Tetragonal	Triclinic
space group	$P\bar{1}$	$I\bar{4}$	$P\bar{1}$
a [Å]	11.3300(8)	18.0642(19)	8.6269(2)
b [Å]	14.9562(9)	18.0642(19)	13.1796(4)
c [Å]	15.8042(8)	10.8324(16)	15.8642(4)
α [°]	109.226(2)	90	82.5080(10)
β [°]	110.945(2)	90	87.0100(10)
γ [°]	94.322(2)	90	80.4920(10)
V [Å^3]	2304.3(2)	3534.8(9)	1762.91(8)
Z	1	2	4
T [K]	100(2)	100(2)	100(2)
wavelength [Å]	0.71073	0.71073	0.71073
ρ_{calcd} [g cm^{-3}]	1.331	1.811	1.658
μ [mm^{-1}]	1.410	2.944	2.598
F(000)	944	1888	872
Θ range [°]	1.493/27.716	2.192/28.678	1.579/28.866
unique reflns	10759	4564	9225
observed reflns	57792	11929	70258
R1[b]/*w*R2(I>2σ)[c]	0.0510/0.1221	0.0334/0.0704	0.0224/0.0500
R1[b]/*w*R2(all data)[c]	0.0745/0.1347	0.0401/0.0773	0.0271/0.0521
GOF[a]	1.037	1.059	1.050

[a]GOF = $[\Sigma w(F_0^2 - F_c^2)^2/(n_0 - n_p)]^{1/2}$. [b]$R_1 = \Sigma(\mathrm{I}\,|F_0| - |F_c|\,\mathrm{I}) / \Sigma\,|F_0|$, $F_0 > 4\sigma(F_0)$. [c]$wR_2 = \{\Sigma[w(F_0^2 - F_c^2)^2/ \Sigma[w(F_0^2)^2]\}^{1/2}$.

Compound **2** crystalizes in a second modification, triclinic in $P\overline{1}$ with the following cell dimensions:
a [Å] = 12.0563(5)
b [Å] = 12.3682(5)
c [Å] = 32.0916(14)
α [°] = 89.0250(10)
β [°] = 80.8230(10)
γ [°] = 89.0510(10)
V [Å^3] = 4722.9(3)

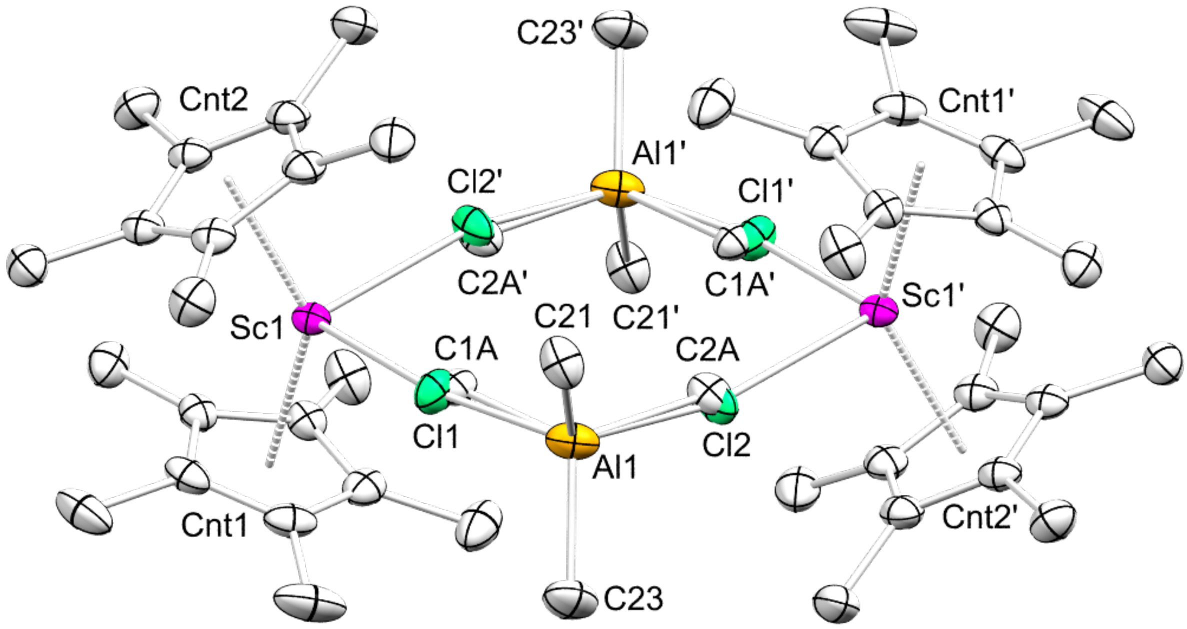

Figure S31. Crystal structure of **2**. Atomic displacement ellipsoids were set at 50% probability. Hydrogen atoms omitted for clarity. A molecule of toluene was omitted for clarity. The crystals were very sensitive to temperatures higher than –30 °C. Selected interatomic distances [Å] and angles [deg]: Sc1−C1A 2.628(18), Sc1−Cl1 2.532(12), Sc1'−Cl2 2.590(9), Sc1'−C2A 2.65(2), Al1−C1A 2.042(19), Al1−Cl1 2.175(12), Al1−C2A 2.08(2), Al1−Cl2 2.165(9), Al1−C21 1.9676(18), Al1−C23 1.970(2), Sc1−C1A−Al1 178.9(8), Sc1−Cl1−Al1 165.8(5), Sc1−C2A'−Al1' 173.3(14), Sc1−Cl2'−Al1' 166.5(6), C1A−Al1−C2A 98.7(7), C1A−Al1−Cl2 100.0(5), Cl1−Al1−Cl2 107.1(4).

'-x, -y, -z

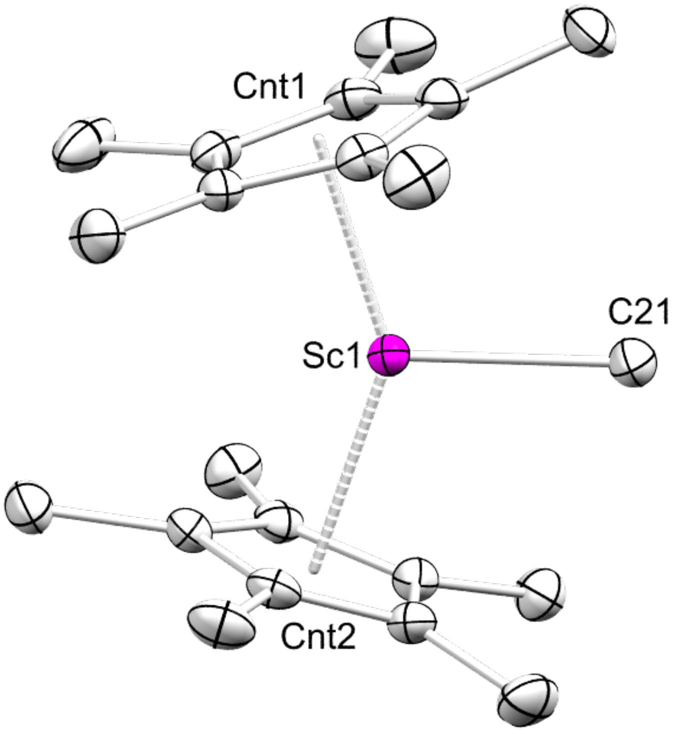

Figure S32. Crystal structure of **3**. Atomic displacement ellipsoids were set at 50% probability. Hydrogen atoms omitted for clarity. Selected interatomic distances [Å]: Sc1−C21 2.266(3), Sc−Cnt1 2.1638(14), Sc1−Cnt2 2.1733(14).

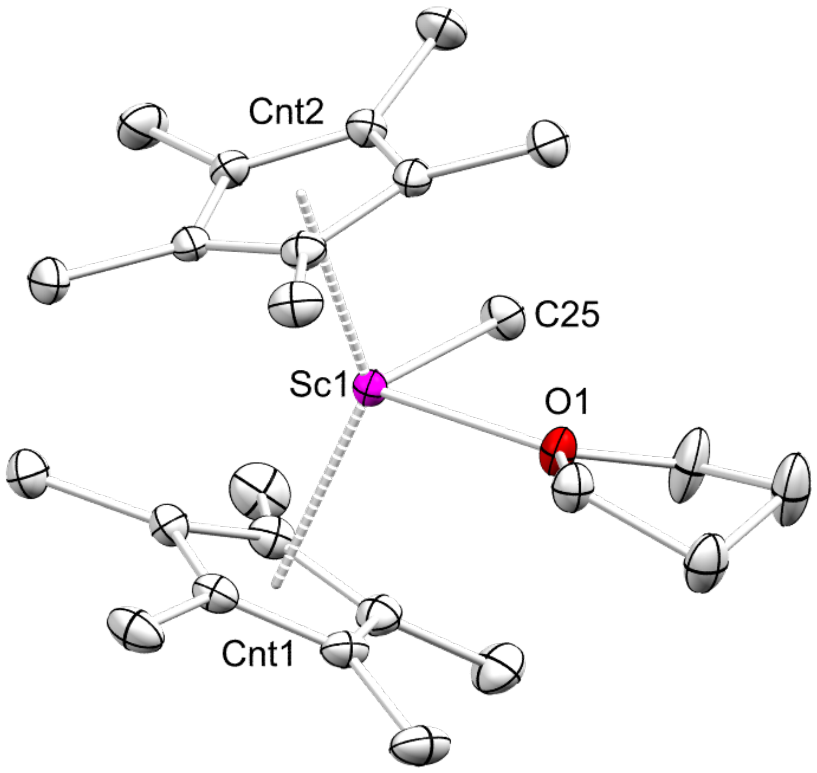

Figure S33. Crystal structure of **3**^{thf}. Atomic displacement ellipsoids were set at 50% probability. Hydrogen atoms and a second molecule of **3** omitted for clarity. Selected interatomic distances [Å] and angles [deg]: Sc1−C25 2.2748(15), Sc1−O1 2.2592(11), Sc1−Cnt1 2.2620(7), Sc1−Cnt2 2.2547(7), O1−Sc1−C25 89.55(5).

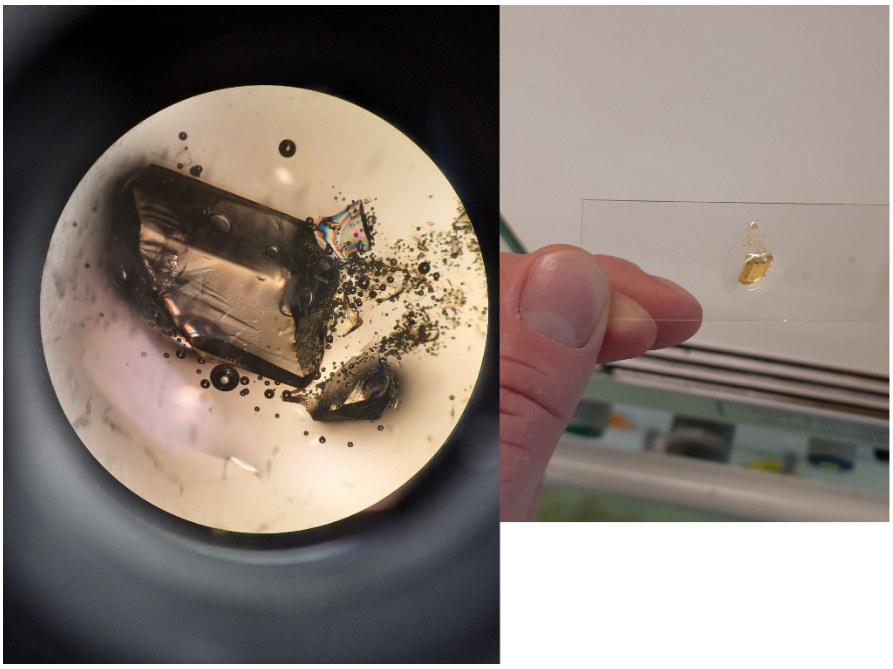

Figure S34. Pictures of crystalline **3**^{thf}.

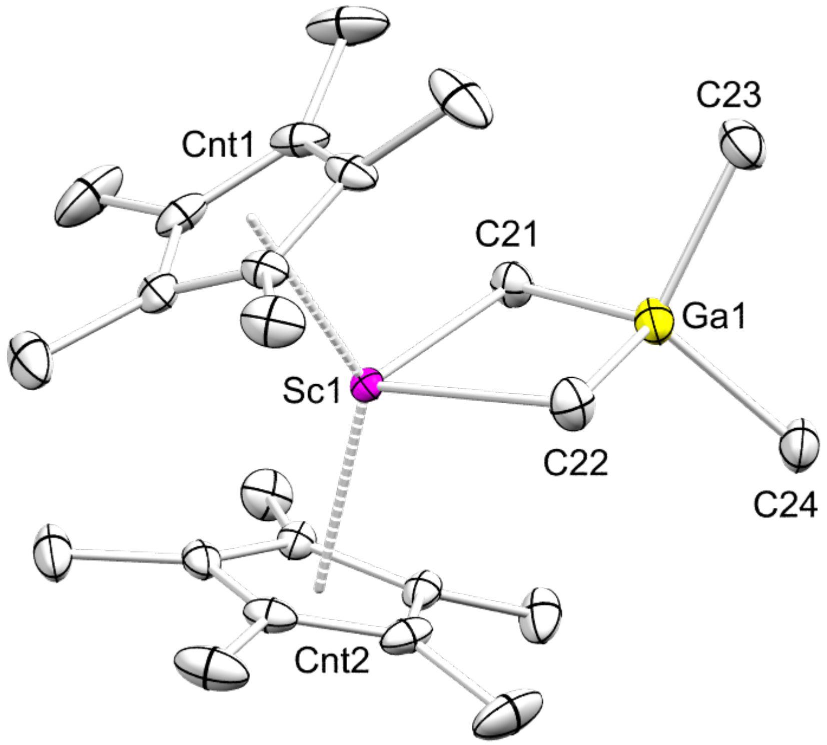

Figure S35. Crystal structure of **1-Ga**. Atomic displacement ellipsoids were set at 50% probability. Hydrogen atoms and a second molecule of **1-Ga** omitted for clarity. Selected interatomic distances [Å] and angles [deg]: Sc1−C21 2.5072(16), Sc1−C22 2.5196(17), Ga1−C21 2.0957(16), Ga1−C22 2.0935(18), Ga1−C23 1.9981(18), Ga1−C24 1.9990(17), Sc1···Ga1 2.9470(3), C21−Sc1−C22 88.46(6), Sc1−C21−Ga1 79.06(5), Sc1−C22−Ga1 78.81(5), C21−Ga1−C22 113.64(7).

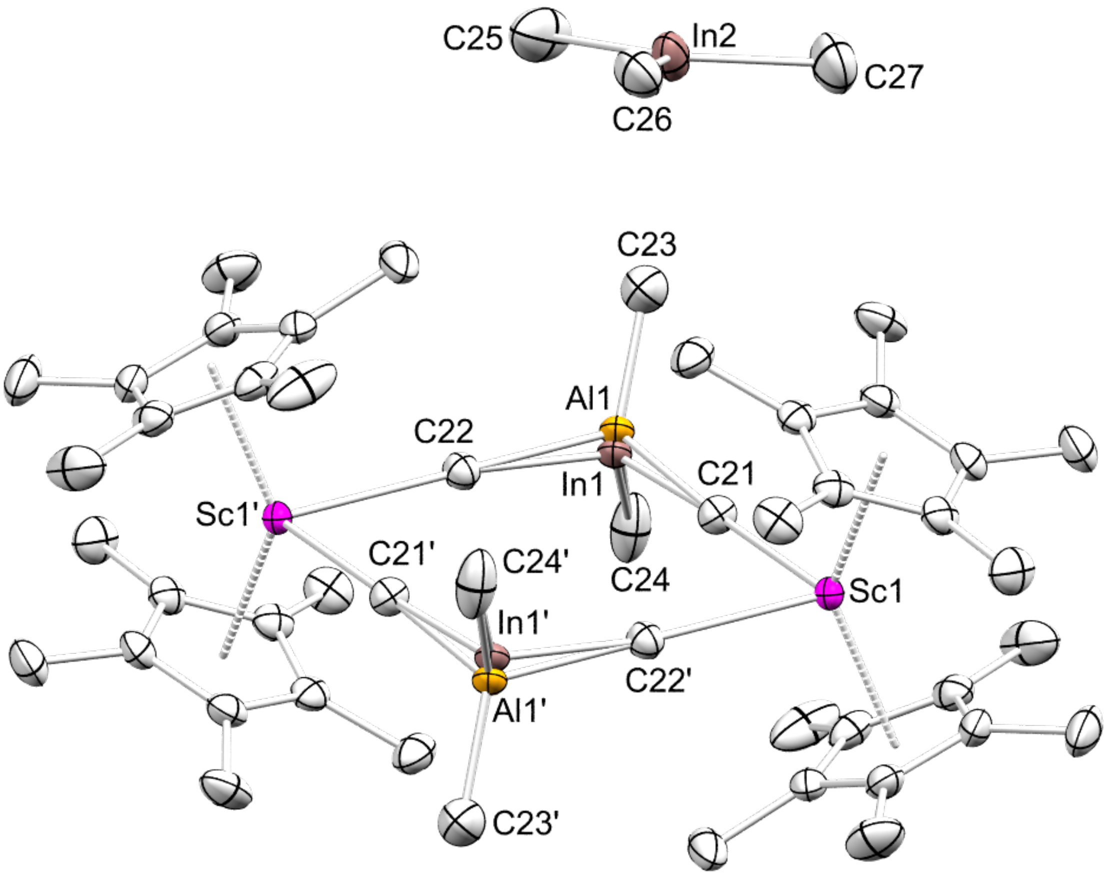

Figure S36. Crystal structure of **1-In**. Atomic displacement ellipsoids were set at 50% probability. Hydrogen atoms omitted for clarity. A molecule of toluene was omitted for clarity. The crystals were very sensitive to air, getting red in seconds even when cooled and covered in lots of oil. Selected interatomic distances [Å] and angles [deg]: Sc1−C21 2.561(2), Sc1−C22’ 2.592(3), In1−C21 2.154(2), In1−C22 2.140(2), In1−C23 2.104(3), In1−C24 1.953(4), Al1−C21 2.215(4), Al1−C22 2.194(4), Al1−C23 1.821(4), Al1−C24 2.147(5), In2−C25 2.166(3), In2−C26 2.171(3), In2−C27 2.159(3), C21−Sc1−C22’ 83.24(7), Sc1−C21−In1 176.32(11), Sc1−C21−Al1 173.33(14), C21−In1−C22 101.75(9), C21−Al1−C22 98.15(17).

‘-x+1, -y+1, -z+1

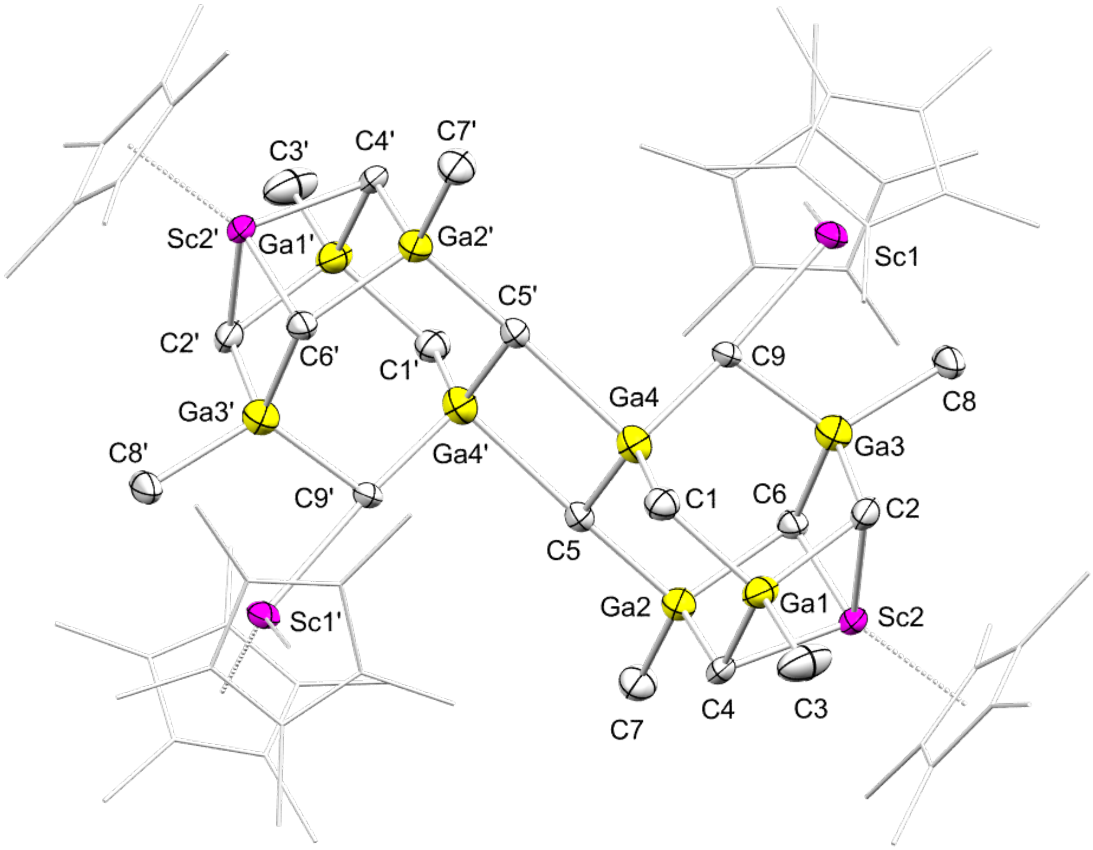

Figure S37. Connectivity crystal structure of **4-Ga**. Atomic displacement ellipsoids were set at 50% probability. Hydrogen atoms omitted for clarity. Two molecules of toluene were omitted for clarity. The Cp* rings are shown in wireframe for clarity.

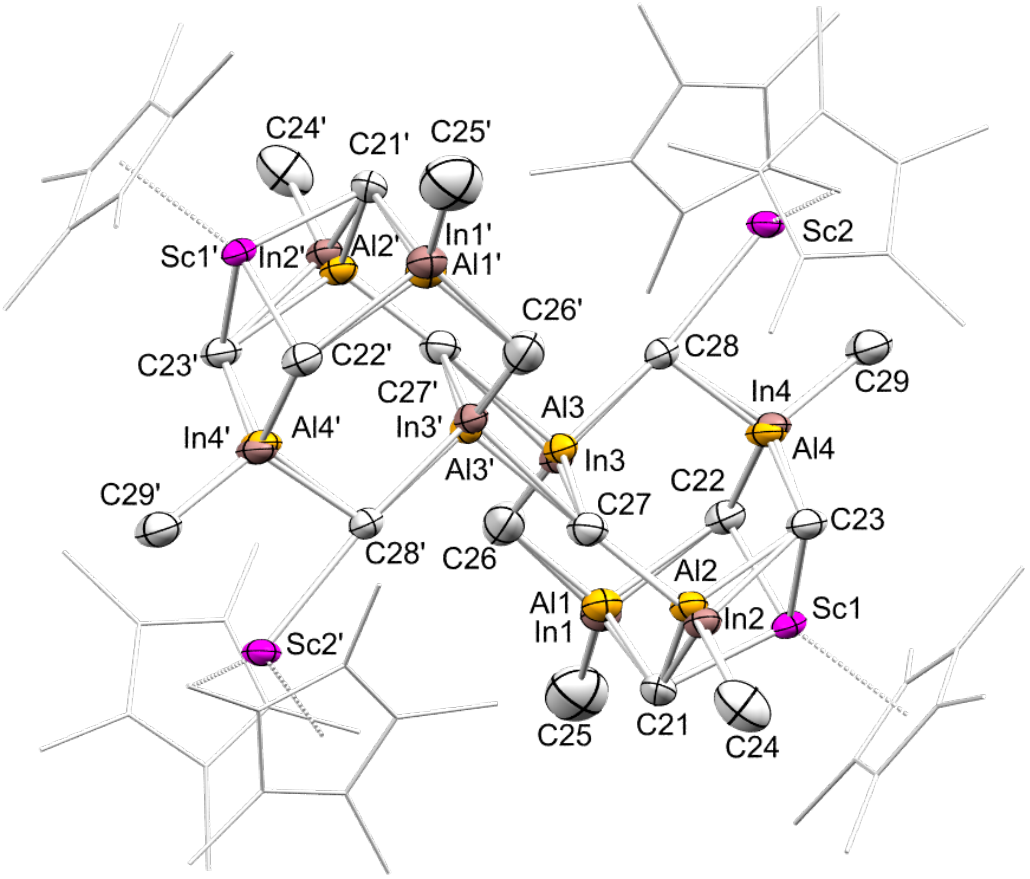

Figure S38. Crystal structure of **4-In**. Atomic displacement ellipsoids were set at 50% probability. Hydrogen atoms and disorders in Cp rings omitted for clarity. The Cp* rings are shown in wireframe for clarity. Selected interatomic distances [Å] and angles [deg]: Sc1−C21 2.230(5), Sc1−C22 2.224(5), Sc1−C23 2.256(5), Sc2−C28 2.445(4), Sc2−C29 2.574(5), In1−C21 2.293(5), In1−C22 2.304(5), In1−C25 2.032(8), In1−C26 2.149(6), Al1−C21 2.122(11), Al1−C22 2.255(12), Al1−C25 2.367(11), Al1−C26 1.953(11), In2−C21 2.241(5), In2−C23 2.277(5), In2−C24 2.078(6), In2−C27 2.242(5), Al2−C21 2.269(17), Al2−C23 2.264(16), Al2−C24 2.316(14), Al2−C27 1.966(13), In3−C26 1.990(7), In3−C27 2.158(5), In3−C27’ 2.489(5), In3−C28 2.199(5), Al3−C26 2.232(14), Al3−C27 2.065(18), Al3−C27’ 2.381(17), Al3−C28 2.106(17), In4−C22 2.10(3), In4−C23 2.12(3), In4−C28 2.05(3), In4−C29 1.97(3), Al4−C22 2.07(4), Al4−C23 2.03(3), Al4−C28 2.03(3), Al4−C29 2.10(3), C21−Sc1−C22 97.39(17), C21−Sc1−C23 97.81(18), C22−Sc1−C23 91.50(17), C28−Sc2−C29 83.95(16), Sc1−C21−In1 81.74(16), Sc1−C21−Al1 81.9(3), Sc1−C22−In1 81.61(16), Sc1−C22−Al1 79.1(3), Sc1−C21−In2 80.87(16), Sc1−C21−Al2 82.4(4), Sc1−C23−In2 79.53(15), Sc1−C23−Al2 81.9(4), Sc1−C22−In4 84.3(7), Sc1−C22−Al4 82.1(8), Sc1−C23−In4 83.1(9), Sc1−C23−Al4 82.1(10), Sc2−C28−In3 173.7(2), Sc2−C28−Al3 169.1(4), Sc2−C28−In4 81.8(7), Sc2−C28−Al4 85.3(8), Sc2−C29−In4 80.0(9), Sc2−C29−Al4 80.8(9).

‘y, -x+1, -z+1

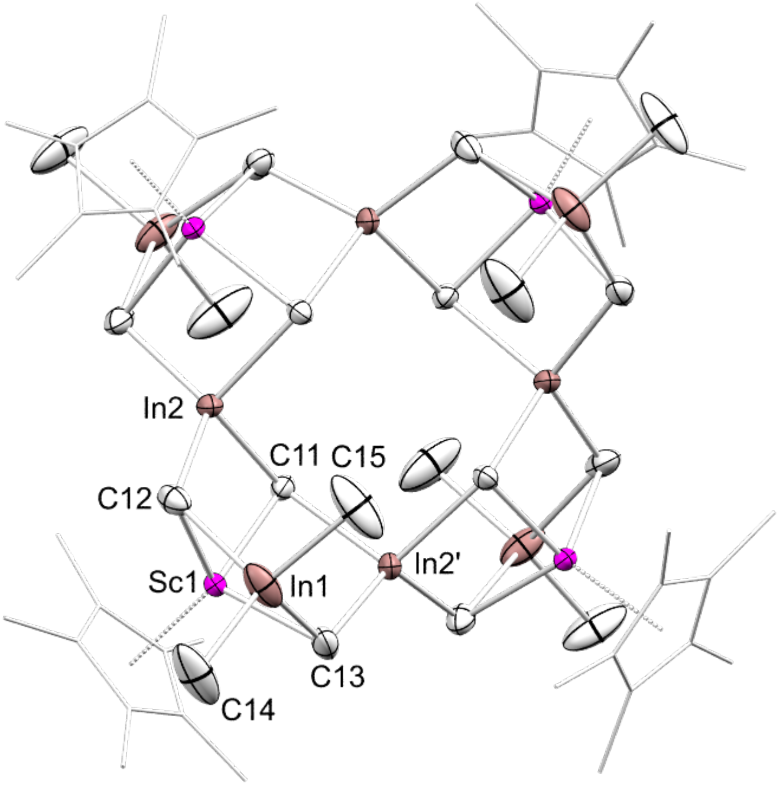

Figure S39. Crystal structure of **5**. Atomic displacement ellipsoids were set at 50% probability. Hydrogen atoms omitted for clarity. The Cp* rings are shown in wireframe for clarity. Selected interatomic distances [Å] and angles [deg]: Sc1−C11 2.306(7), Sc1−C12 2.255(8), Sc1−C13 2.265(8), In1−C12 2.346(10), In1−C13 2.340(9), In1−C14 2.184(9), In1−C15 2.174(11), In2−C11 2.275(7), In2−C12 2.250(8), In2'−C11 2.266(7), In2'−C13 2.254(8), C11−Sc1−C12 98.5(3), C11−Sc1−C13 97.2(3), C12−Sc1−C13 100.0(3), Sc1−C12−In1 80.5(3), Sc1−C13−In1 80.5(3), Sc1−C11−In2 79.1(2), Sc1−C12−In2 80.7(3), Sc1−C11−In2' 79.5(2), Sc1−C13−In2' 80.6(3).

'y, -x+1, -z+1

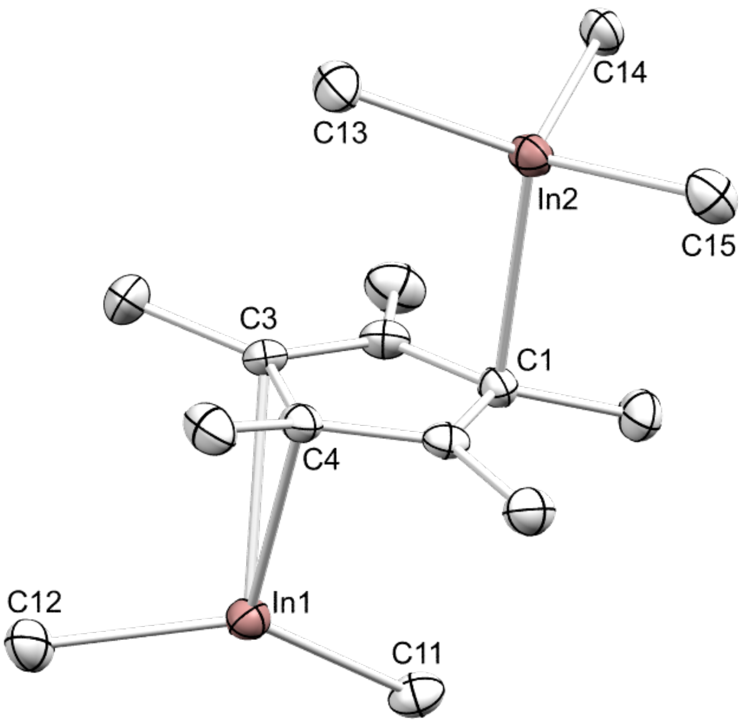

Figure S40. Crystal structure of **6**. Atomic displacement ellipsoids were set at 50% probability. Hydrogen atoms and a second molecule of **6** omitted for clarity. Selected interatomic distances [Å] and angles [deg]: In1−C3 2.561(2), In1−C4 2.407(2), In1−C11 2.142(2), In1−C12 2.154(2), In2−C1 2.590(2), In2−C13 2.164(2), In2−C14 2.235(2), In2−C15 2.180(2), C11−In1−C12 137.74(10), C11−In1−C3 108.51(8), C12−In1−C3 100.60(9), C11−In1−C4 114.65(8), C12−In1−C4 106.70(9), C13−In2−C14 113.10(10), C13−In2−C15 123.95(10), C13−In2−C1 109.53(8), C14−In2−C15 106.71(9), C14−In2−C1 98.23(8), C15−In2−C1 101.75(9).
